# Environmental health influences in pregnancy and risk of gestational diabetes mellitus: a systematic review

**DOI:** 10.1186/s12889-022-13965-5

**Published:** 2022-08-18

**Authors:** Claudia Eberle, Stefanie Stichling

**Affiliations:** Medicine With Specialization in Internal Medicine and General Medicine, Hochschule Fulda, University of Applied Sciences, Leipziger Strasse 123, 36037 Fulda, Germany

**Keywords:** Gestational diabetes, Environmental health, Environmental risk factor, Pollutants, Systematic review

## Abstract

**Background:**

Gestational diabetes mellitus (GDM) is one of the most common pregnancy complications globally. Environmental risk factors may lead to increased glucose levels and GDM, which in turn may affect not only the health of the mother but assuming hypotheses of "fetal programming", also the health of the offspring. In addition to traditional GDM risk factors, the evidence is growing that environmental influences might affect the development of GDM. We conducted a systematic review analyzing the association between several environmental health risk factors in pregnancy, including climate factors, chemicals and metals, and GDM.

**Methods:**

We performed a systematic literature search in Medline (PubMed), EMBASE, CINAHL, Cochrane Library and Web of Science Core Collection databases for research articles published until March 2021. Epidemiological human and animal model studies that examined GDM as an outcome and / or glycemic outcomes and at least one environmental risk factor for GDM were included.

**Results:**

Of *n* = 91 studies, we classified *n* = 28 air pollution, *n* = 18 persistent organic pollutants (POP), *n* = 11 arsenic, *n* = 9 phthalate *n* = 8 bisphenol A (BPA), *n* = 8 seasonality, *n* = 6 cadmium and *n* = 5 ambient temperature studies. In total, we identified two animal model studies. Whilst we found clear evidence for an association between GDM and air pollution, ambient temperature, season, cadmium, arsenic, POPs and phthalates, the findings regarding phenols were rather inconsistent. There were clear associations between adverse glycemic outcomes and air pollution, ambient temperature, season, POPs, phenols, and phthalates. Findings regarding cadmium and arsenic were heterogeneous (*n* = 2 publications in each case).

**Conclusions:**

Environmental risk factors are important to consider in the management and prevention of GDM. In view of mechanisms of fetal programming, the environmental risk factors investigated may impair the health of mother and offspring in the short and long term. Further research is needed.

**Supplementary Information:**

The online version contains supplementary material available at 10.1186/s12889-022-13965-5.

## Background

Gestational diabetes mellitus (GDM) is one of the most common pregnancy complications worldwide [1]. It is defined as any degree of glucose intolerance with onset or first recognition during pregnancy [1]. In the past 20 years, the prevalence of GDM has increased [1–4], so that up to ca. 14% of all pregnancies worldwide are complicated by GDM [5]. The association between GDM and adverse pregnancy maternal and neonatal outcomes like preeclampsia, caesarean section, preterm birth, macrosomia and neonatal hypoglycaemia, is well known [5, 6]. In addition, the evidence of long-term health impacts on mothers and offspring is increasing [7]. For example, there is a growing risk for the mother of developing type 2 diabetes (T2D) [8, 9] and cardiovascular disease (CV) [10, 11] in later life. However, there is an increased risk for offspring to develop obesity and abnormal glucose metabolism in mid-childhood [1, 6, 12]. This suggests that metabolic and cardiovascular conditions in the offspring may be associated with mechanisms of perinatal (or fetal or transgenerational) programming attributable to genomic and environmental influences of the mother during perigestation [13].

The “Thrifty Phenotype Hypothesis” suggests that physiological and metabolic adaptations may be induced, for example, by fetal malnutrition [14]. During critical periods of pregnancy, the nutrient supply of some organs is limited to ensure the supply of the brain or the heart. This may lead to a permanent programmed metabolism of the fetus which results in harmful long-term consequences triggered by nutritional abundance in later life [14]. Studies found an association between exposure to longer starving periods in utero and reduced glucose tolerance in adulthood [15], and between low birth weight and adult glucose and insulin metabolism [16].

The mechanisms behind the Thrifty Phenotype Hypothesis are diverse [13]: the genotype of the fetus may be influenced by mutations which result in impaired insulin secretion or in polymorphisms [17, 18]. Adverse intrauterine factors such as maternal food restriction, malnutrition, stress, placental dysfunction, obesity, gestational diabetes, hypertension, preeclampsia, and gestational weight gain are very important influences. Adverse postnatal environmental factors like malnutrition, inactivity or aging also play an important role [13].

The “oxidative stress” hypothesis is one other possible mechanism. In the sensitive state of pregnancy, the metabolic state can be easily disturbed, which results in dysblances towards oxidative stress, leading to insulin resistance, gestational diabetes, and gestational hypertension [19–21]. There seems to be a relation between higher rates of malformations and anomalies in offspring of diabetic mothers and mechanisms of oxidative stress, hyperglycemia-induced ROS, mitochondrial dysfunction and altered glycosylation [22–24].

Furthermore, according to Gluckman et al. and the “Predictive Adaptive Responses Hypothesis”, unfavourable environmental conditions induced by diseases such as GDM can lead to adapted developmental processes in utero [13, 14, 25]. Another hypothesis called the “Fetal Insulin Hypothesis” points out that genetically determined alterations within the insulin signaling cascades could be a reason for altered fetal development as well as long-term metabolic changes [14]. This hypothesis is supported by studies which showed that genetic influences like polymorphisms or mutations, and environmental effects on the fetus, which can result in altered glucose tolerance and insulin resistance, are strongly related to long-term consequences like metabolic and cardiovascular diseases in later life [14, 22, 26].

It is of great interest to better characterize the potential environmental risk factors for GDM and its ability to “program” short- and long-term consequences in offspring. The aetiology of GDM is multifactorial [27]. “Traditional” risk factors such as excess caloric consumption, lack of physical activity and increased BMI [28] do play an essential role, but evidence is growing that environmental exposures such as air pollution [29, 30], climate factors [31, 32] and endocrine disrupting chemicals [33–35] and metals [36, 37] affect the development of GDM as well. Therefore we aimed at performing a systematic review analyzing the association between environmental health risk factors in pregnancy, including climate factors, chemicals and metals, and gestational diabetes or adverse glycemic parameters. We provide an overview of potential environmental risk factors for GDM, considering a wide range of these factors including epidemiological human and animal model studies.

## Methods

### Search strategy

We conducted a systematic literature search in Medline (PubMed), EMBASE, CINAHL, Cochrane Library and Web of Science Core Collection databases for research articles published until March 2021. Keywords (gestational diabetes, bisphenol A, phthalate, persistent organic pollutants, cadmium, arsenic, air pollution, ambient temperature, season, air humidity) were selected from the Medical Subject and Embase Subject Headings and searched as titles/abstracts terms (Additional file 1). In general, the steps of literature search were:

1. Systematic search in databases.
2. Elimination of duplicates.
3. Screening of titles and abstracts and selection of studies.
4. Appraisal of selected studies for eligibility.
5. Selection of suitable studies and classification of these studies according to risk factors.
6. Extraction and synthesis of relevant data.

### Study selection

Peer-reviewed epidemiological human studies and animal model studies with full-texts in English and German were included. We considered studies that examined GDM as an outcome and/or maternal glycemic outcomes and at least one environmental risk factor for GDM. In general, we included papers that analyzed the following environmental risk factors, as there were the most frequently investigated health influences: bisphenol A (BPA), phthalate, persistent organic pollutants (POP), cadmium, arsenic, air pollution, ambient temperature, season and air humidity.

Duplicates, other study designs (e.g. reviews, meta-analyses, case-reports), letter, comments, notes, editorials and conference proceedings were excluded. In addition, we excluded papers that analyzed GDM treatment methods, papers that focussed on women with pre-existing type 1 or type 2 diabetes, and publications that examined other pregnancy outcomes, for example gestational hypertension, preeclampsia, macrosomia and preterm birth. The PRISMA flow chart is provided in the Additional file 1.

### Data synthesis and analysis

We extracted authors, year of publication, location, study design, sample sizes, characteristics of the subjects, methods, outcomes of interest, study limitations, diagnostic criteria for GDM, main findings and statistical calculations. Furthermore, we classified the publications according to the environmental risk factors and the outcomes (GDM vs. glycemic parameters).

## Results

Our systematic literature research resulted in 819 articles (see Additional file 1). After excluding unsuitable articles and duplicates (*n* = 514), *n* = 105 full-text articles were assessed for eligibility based on our inclusion and exclusion criteria. In total, *n* = 91 studies were analyzed in this systematic review. We identified *n* = 28 air pollution, *n* = 18 POP, *n* = 11 arsenic, *n* = 9 phthalate *n* = 8 BPA, *n* = 8 season, *n* = 6 cadmium and *n* = 5 ambient temperature studies. We found no studies examining the association between air humidity and GDM and only *n* = 2 animal model studies.

The majority of studies were conducted in China (*n* = 32) and in the USA (*n* = 25). Five studies took place in Taiwan, *n* = 4 in UK, *n* = 4 in Canada, *n* = 4 in Australia, *n* = 3 in Iran, *n* = 2 in Spain, *n* = 2 in Greece, *n* = 2 in France, *n* = 2 in Sweden, *n* = 1 in Guadeloupe, *n* = 1 in Argentina, *n* = 1 in Chile, *n* = 1 in Japan, *n* = 1 in Israel and *n* = 1 in Denmark. Most studies were prospective cohort studies (*n* = 41) and retrospective cohort studies (*n* = 16). 13 publications were case–control studies and 12 were cross-sectional. 4 papers were conducted as longitudinal birth cohort studies, *n* = 2 were interventional studies, *n* = 2 population based cohort studies and *n* = 2 were semi-ecological-studies. Table 1 gives an overview of the included studies.

**Table 1:** Overview of included studies

**Environmental risk factor**	**Number of studies**
Air pollution	28
Persistent organic pollutants (POP)	18
Arsenic	11
Phthalate	9
Seasonality	8
Bisphenol A (BPA)	8
Cadmium	6
Ambient temperature	5
**Locations**	**Number of studies**
China	32
USA	25
Taiwan	5
UK	4
Canada	4
Australia	4
Iran	3
Spain	2
Greece	2
France	2
Sweden	2
Guadeloupe	1
Argentina	1
Chile	1
Japan	1
Israel	1
Denmark	1

### GDM screening methods

Across the human studies, different methods of GDM screening and diagnosis were used. The most frequent screening methods involved a one-step approach where GDM is diagnosed based on results of a single fasting oral glucose tolerance test (OGTT), as well as a two-step approach where women are pre-screened, generally with a non-fasting glucose challenge test (GCT). In some studies GDM was self-reported. Five studies also evaluated various markers of insulin resistance (IR) for example the Matsuda index and the homeostasis model assessment of insulin (HOMA-IR, HOMA S) and beta cell function like insulinogenic index (IGI)/HOMA-IR, and HOMA B.

### Environmental risk factors

#### Phenols and GDM (*n* = 5 studies)

Overall, the studies showed no clear and rather heterogenic results [38–42]. Hou et al. (*n* = 390 [38] and Zhang et al. (*n* = 1841) [42]) indicated significant associations (*p* < 0.01) for BPAF and 2-t-OP, whereas two prospective cohort studies (*n* = 535 [41], *n* = 620 [40]) and one case–control-study (*n* = 22 cases, 72 controls [39]) did not observe clear associations between BPA and GDM. In a cohort-study by Wang et al., the odds of GDM were even reduced by 27% (OR = 0.73; 95% CI = 0.56, 0.97) [40].

#### Phenols and glycemic outcomes (*n* = 4)

All *n* = 4 studies reported positive associations between glycemic outcomes and phenols in exposed women [38, 41, 43, 44]. Three studies, [(*n* = 350 [43], *n* = 245 [44], *n* = 535 [41]) found significant associations between BPA concentrations and glucose levels (*p* < 0.05 [43], *p* < 0.01 [44], *p* < 0.05 [41]). However, Bellavia et al. observed this association only on obese and overweight women [43].

#### *Pthalates and GDM (n* = *6)*

An animal model study performed on rats (*n* = 24) showed significant positive associations for the progression of GDM through Di-n-butyl phthalate (*p* > 0.05 [45]).

Consistent with this, 3 of 5 human studies observed positive associations between certain phthalates (mono-iso-butylphthalate, mono-n-butylphthalate, mono-2-ethyl-5-oxohexyl phthalate (*p* < 0.05) [46], monoethyl phthalate (95% CI:1.61 (1.10, 2.36) [47], mono-(2-ethylhexyl) phthalate (*p* = 0.019) [48]) and GDM. Two cohort studies by Shapiro e al. (*n* = 1274 [49], *n* = 1795 [50]) did not find significant associations between phthalates (aOR third quartile = 2.0, 95% CI = 0.9–4.4; aOR fourth quartile = 2.0, 95% CI = 0.9–4.8) [49] or triclosan (aOR third quartile = 0.9, 95% CI (0.3–2.5) aOR fourth quartile = 0.9, 95% CI (0.4–2.5) [50] and GDM.

#### Phthaltes and glycemic outcomes (*n* = 7)

Most studies (*n* = 4) showed positive associations between phthalate exposure and adverse glycemic outcomes (*p* < 0.01 [48], *p* < 0.05 [47, 51, 52]. James Todd et al. (*n* = 350) showed that second trimester mono-ethyl phthalate was associated with increased odds of impaired glucose tolerance (adj. OR: 7.18; 95% CI: 1.97, 26.15). In contrast, *n* = 3 publications (*n* = 72 [53], *n* = 1274 [49], *n* = 1795 [50]) observed not significant associations and concluded that phthalate exposure might not be associated with adverse glycemic values.

#### *POPs and GDM (n* = *15)*

The majority of the papers found that POPs exposure obviously increased the risk for GDM [35, 54–67]. In general, *n* = 10 out of *n* = 15 studies reported significant positive associations (*p* < 0.05 [55, 57–60, 62, 64, 67], *p* < 0.001 [63], *p* < 0.01 [66]) for POPs like PCBs [55, 60, 63, 67], PBDEs [55, 59, 62], EtP [57], PCAs [58], PFASs [66] and OCs [64]. In contrast, *n* = 4 studies found no significant associations between POPs and GDM [35, 56, 61, 65]. Alvarez-Silvares et al. [54] reported an inverse relationship between PBDE and PCB levels in placenta and GDM.

#### POPs and glycemic outcomes (*n* = 5)

All studies showed that POPs had clear negative effects on glycemic parameters in pregnant women [35, 58, 65, 68, 69]. In total, *n* = 3 of *n* = 5 studies reported that POPs (PB [68], PFCAs [58], PFAS [65]) resulted in elevated blood glucose levels (*p* < 0.05 [58, 65, 68]. Shapiro et al. (*n* = 1274) observed elevated odds of gestational IGT ((OR = 3.5, 95% CI = 1.4–8.9) [35]. Wang et al. (*n* = 217) found time-specific inverse associations (*p* < 0,05) between BP-3 and pregnancy glucose levels [69].

#### *Arsenic and GDM (n* = *10)*

One animal model study by Bonventura et al. (rats) noted clearly that arsenic alters glucose homeostasis during pregnancy by altering beta-cell function, increasing the risk of developing gestational diabetes (*p* < 0.05) [70]. Overall, most human studies (*n* = 8 out of *n* = 9) found a positive relation between exposure to arsenic and GDM (*p* = 0.04 [71], *p* < 0.05 [37, 72–75], *p* < 0.001 [76], *p* = 0.37 [77]). Two of these studies [72, 73] found this association only in obese and overweight women. Contrary to this, Muñoz et al. reported no significant association between GDM and inorganic arsenic exposure tertiles [78]).

#### Arsenic and glycemic outcomes (*n* = 2)

Ettinger et al. (*n* = 532) reported a significant association for arsenic exposure and increased risk for impaired glucose tolerance (*p* = 0.008 [79]), whereas Farzan et al. (*n* = 1151) found no significant association [72].

#### *Cadmium and GDM (n* = *6)*

In general, the majority of studies (*n* = 4 of *n* = 6) found significant positive associations for cadmium exposure and GDM (*p* = 0.003 [80], *p* = 0.03 [81], *p* < 0.05 [36, 82]). However, Oguri et al. found no association for elevated blood cadmium with increased GDM risk [83] and Romano et al. only found a slight association in women with normal weight (OR = 1.32, 95% CI 0.88–1.98) [84].

#### Cadmium and glycemic outcomes (*n* = 2)

Romano et al. observed no significant association for glucose intolerance (OR = 1.11, 95%CI 0.85–1.45) and Soomro et al. reported that cadmium was statistically related to having had a diagnosis of IGT (aOR = 1.61, 95% CI 1.05–2.48) [36].

#### *Season and GDM (n* = *7)*

In general, most studies (*n* = 5 of *n* = 7) found clear seasonal variations in the prevalence of GDM. Strong relations were reported for GDM prevalence in spring and summer in *n* = 5 studies (*p* < 0.0001 [85] *p* = 0.027 [86], *p* = 0.01 [32], *p* < 0.001 [87], *p* = 0.02 [88]). However, Shen et al. and Petry et al. (*n* = 2120 [89], *n* = 1074 [90]) observed no significant associations with seasonality (*p* = 0.51 [89], *p* = 0.4 [90]).

#### Season and glycemic outcomes (*n* = 6)

In total, *n* = 5 of *n* = 6 studies outlined significant seasonal trends, with higher glucose levels in summer (*p* < 0.0001 [32, 85], *p* = 0.009 [89], *p* < 0.001 [91], *p* < 0.05 [88]). In contrast, Petry et al. revealed no significant association of OGTT fasting glucose concentrations and seasonality [amplitude: 21.3 (− 24.1, 66.6) [90]).

#### Ambient temperature and GDM (*n* = 5)

All publications found that rising environmental temperature was distinctly associated with an increased risk of GDM [31, 92–95]. Furthermore, a large cohort study by Zhang et al. reported that extreme low temperature exposure increased the risk of GDM as well (*p* < 0.05 [95]).

#### Ambient temperature and glycemic outcomes (*n* = 2)

Retnajaran et al. (*n* = 318) indicated that rising environmental temperature in the 3– 4 weeks prior to glucose tolerance testing in pregnancy was independently associated with maternal beta cell dysfunction and blood glucose levels [92] and Vasileiou et al. noted that temperatures above 25 °C might lead to increased glucose levels [94].

#### *Air pollution and GDM (n* = *25)*

In summary, *n* = 22 of *n* = 25 studies showed that air pollution (through PM_2.5_ [29, 96–107], NO [30, 108–112], NO_2_ [30, 108, 109, 112–114], CO [30, 113], PM_10_ [98, 100, 104], BC [98], SO_2_ [30, 100, 103, 104, 115], O_3_ [30]) was clearly associated with an increased risk of GDM. Eight studies found a significant association (*p* < 0.01 [107, 109], *p* < 0.05 [103, 110, 111, 113], (aOR = 1.69 (1.41, 2.03) [108]). However, Fleisch et al. (*n* = 159.373) found higher odds of GDM in women less than 20 years old (95% CI: 1.08, 1.70) [116], whereas Padula et al. in a cross-sectional-study (*n* = 262.182) reported consistent inverse associations between exposure to air pollution during the first two trimesters and GDM (*p* < 0.01) [117].

#### Air pollution and glycemic outcomes (*n* = 9)

Overall, the most articles pointed out that exposure to air pollution (through PM_2.5_ [98–100, 105, 107, 118–121], PM_10_ [98, 100, 119, 121], PM_1_ [121], BC [98], NO_2_ [119], CO [119], SO_2_ [100]) was able to increase blood glucose levels in pregnant women. In *n* = 7 of *n* = 9 studies, air pollution was significantly associated (*p* < 0.05 [98, 99, 105, 119, 120], *p* < 0.01 [107, 121]) with elevated fasting blood glucose concentrations. The remaining *n* = 2 studies by Fleisch et al. (*n* = 2.093) [118] and Lin et al. (*n* = 12.842) [100]) also found associations, but they were not significant.

## Discussion

In this systematic review, the current evidence assessing environmental risk factors and GDM has been analyzed. Whilst we found clear evidence for an association between GDM and air pollution [29, 96–107], ambient temperature [31, 92–95], season [32, 85–88], cadmium [36, 80–82], arsenic [37, 70–77], POPs [55, 57–60, 62–64, 66, 67] and phthalates [45–48], the findings regarding phenols were rather heterogeneous. The results for adverse glycemic outcomes also showed clear associations regarding air pollution [98–100, 105, 107, 118–121], ambient temperature [92, 94], season [32, 85, 88, 89, 91], POPs [35, 58, 65, 68], phenols [38, 41, 43, 44] and phthalates [47, 48, 51, 52]. Our findings regarding cadmium and arsenic were inconsistent with only *n* = 2 publications in each case.

The results suggest that environmental health influences such as bisphenol A (BPA), phthalate, persistent organic pollutants (POPs), cadmium, arsenic, air pollution, ambient temperature and season might be associated with an increased risk for GDM and with adverse glycemic values. These results suggest that environmental risk factors are important to consider in the management and prevention of GDM, especially in the context of the growing dissemination of environmental pollutants and toxins. In view of key theses on fetal (or transgenerational) programming, which were mentioned at the beginning, the environmental factors examined may impair the health of mother and offspring in the short and long-term and lead to the development of diseases. Furthermore, the mechanisms underlying the potential correlation of environmental influences with GDM remain obscure and need more research. However, in previous research, it was found that *air pollution* exposure during the second trimester was significantly associated with GDM. Substances like SO_2_, oxynitride (NOX, NO_2_, NO), CO, and O_3_ all showed a linear trend effect on GDM [30]. On the other hand, the data on fine particular matter (PM) is inconsistent. Some research studies report that greater exposure to fine particulate matter and other traffic-related pollutants during pregnancy was not associated with GDM [118]. Other studies observed that PM in the 2nd trimester were associated with higher odds of GDM [29].

Moreover, an association between *ambient temperature / seasonality* and type 1 and/or type 2 diabetes was found in meta-analyses and observational studies [122–124]. There was discussion about whether this association could be transferred to GDM. Very cold or very high ambient temperature may lead to improved insulin sensitivity, due to activation of brown adipose tissue [125], or to spurious increased blood glucose concentrations, because of dehydration and hemoconcentration [91]. Another systematic review also found that the seasonality of GDM was consistent across studies, with higher prevalence of GDM generally observed in the summer months and with clear associations between ambient temperature and GDM [126]. Future studies need more consistency in exposure and outcome assessment and diverse study populations need to be included to identify potential high-risk population subgroups.

Furthermore, other studies also suggest that *chemical* exposure has a negative effect on women’s health [127, 128]. Chemicals like polychlorinated biphenyls (PCBs), perfluoroalkyl substances (PFAS), polybrominated diphenyl ethers (PBDEs), BPA and some pesticides currently in use are widespread in consumer and personal care products or food, yet have an endocrine disrupting property which can lead to adverse pregnancy outcomes such as GDM [129, 130]. Maternal susceptibility for developing GDM might be increased through chemicals that disrupt or damage pancreatic β cells and environmental chemicals that interfere with the peroxisome proliferator-activated receptor signalling pathway, which mediates placental development and is fundamental to lipid metabolism [131, 132]. Some animal and human studies also indicated that endocrine disrupting chemicals are diaplacental [133–136] and thus result in adverse pregnancy outcomes like fetal growth restriction or preterm birth [134, 135]. Even though endocrine disrupting chemicals seem to have an extensive impact on women’s and offspring’s health, the data on their effect on GDM is still limited.

Besides, *metals* can persist in the environment and some heavy metals such as cadmium have biological half-lives of more than ten years. Therefore, they have been a public health concern for many years [137]. Metals including arsenic and cadmium are classified as endocrine disrupting substances because they seem to have estrogenic activity [138]. High level of arsenic in maternal blood or meconium might be associated with increased risk of GDM [75, 76]. Other metals like nickel, antimony, cobalt or vanadium also showed positive associations with an increased risk of GDM [37]. Just like endocrine disrupting chemicals, metals are easily transported through the placenta [139, 140] and might cause adverse pregnancy outcomes like adverse. glycemic parameters, small for gestational age and stillbirths [140–142].

### Limitations

In some cases and depending on the risk factors, the sample sizes were small, which is a limitation as there was limited statistical significance. With regard to arsenic and cadmium and glycemic values, we were able to identify only very few studies. In general, further studies are required for all risk factors examined in order to reproduce the results. Furthermore, we included studies that are observational in nature and therefore cannot confirm whether the observed association is causal. Potential confounders, such as family history of diabetes, diet, physical activity and other exogenous compounds with similar exposure sources were mostly not measured and considered, but might influence the findings. Probably the major limitation of the manuscript is the lack of clear evidence and transfer related to clinical consultation. Nevertheless, it is important to point out a possible overarching relationship. More research needs to be done.

## Conclusion

This systematic review found that environmental health influences such as bisphenol A (BPA), phthalate, persistent organic pollutants (POP), cadmium, arsenic, air pollution, ambient temperature and seasonality might be associated with an increased risk of GDM. Therefore, environmental risk factors must be considered in the prevention and management of GDM – in clinical practice and research.

Metabolic conditions in offspring might be a result of fetal programming due to maternal perigestational genomic and environmental conditions. As far as fetal programming is concerned, environmental health influences can impair the health of mother and child during pregnancy and later in life. Our findings need to be replicated in other studies. There is a need for further research in particular with regard to the association between GDM and phenols and with regard to glycemic values and arsenic and cadmium. Furthermore, we identified very few animal studies and none on humidity and GDM. Overall, more research is required to analyze this increasingly important research topic in more detail. Besides, the mechanisms underlying the potential correlation of environmental influences with GDM need to be analyzed.

## Supplementary Information

**Additional file 1.**

## Data Availability

All data are included within the article and its supplementary information files.
