# Supplementary material for: Environmental health influences in pregnancy and risk of gestational diabetes mellitus: a systematic review
Source: BMC Public Health. 2022 Aug 18;22:1572. doi: 10.1186/s12889-022-13965-5 (PMC9389831; doi:10.1186/s12889-022-13965-5)
Supplement: Supplementary file 1 — Additional file 1. [file 12889_2022_13965_MOESM1_ESM.docx]

**SUPPLEMENTARY INFORMATION**

**Environmental health influences in pregnancy and risk of gestational diabetes mellitus: A systematic review**

Claudia Eberle, M.D.^1^, Stefanie Stichling, M.Sc.^1^

**Table of Contents**

Search strategies page 2

PRISMA flow chart page 3

Overviews of included studies pages 4-24

**Table 1: Search strings.**

| **Database** | **Medline (PubMed)** | **Embase** | **Cochrane** | **CINAHL** | **Web of Science Core Collection** |
| --- | --- | --- | --- | --- | --- |
| **Search** | (gestational diabetes[MeSH Terms]) AND ((bisphenol a glycidyl methacrylate[MeSH Terms]) OR (acids, phthalic[MeSH Terms]) OR (cadmium[MeSH Terms]) OR (arsenic[MeSH Terms]) OR (air pollution[MeSH Terms]) OR (ambient temperature[Title/Abstract]) OR (affective disorder, seasonal[MeSH Terms]) OR (air humidity[Title/Abstract]) OR (persistent organic pollutants[Title/Abstract])) | ´pregnancy diabetes mellitus`/exp AND ('4,4` isopropylidenediphenol'/exp OR 'phthalic acid'/exp OR 'persistent organic pollutant'/exp OR 'arsenic'/exp OR 'cadmium'/exp OR 'air pollution'/exp OR 'environmental temperature'/exp OR 'season'/exp OR 'air humidity'/exp) | (MeSH descriptor: [Diabetes, Gestational] explode all trees) AND (MeSH descriptor: [Bisphenol A-Glycidyl Methacrylate] explode all trees OR MeSH descriptor: [Phthalic Acids] explode all OR MeSH descriptor: [Persistent Organic Pollutants] explode all trees OR MeSH descriptor: [Arsenicals] in all MeSH products OR MeSH descriptor: [Air Pollution] in all MeSH products OR ("ambient temperature"):ti,ab,kw OR MeSH descriptor: [Seasons] explode all trees OR (air humidity):ti,ab,kw) | ((TI gestational diabetes) OR (AB gestational diabetes)) AND (((TI bisphenol a) OR (AB bisphenol a)) OR ((TI phthalate) OR (AB phthalate)) OR ((TI cadmium) OR (AB cadmium)) OR ((TI arsenic) OR (AB arsenic)) OR ((TI persistent organic pollutants) OR (AB persistent organic pollutants)) OR ((TI air pollution) OR (AB air pollution)) OR ((TI ambient temperature) OR (AB ambient temperature)) OR ((TI seasons) OR (AB seasons)) OR ((TI air humidity) OR (AB air humidity))) | ((gestational diabetes) AND ((bisphenol a) OR (phthalate) OR (cadmium) OR (arsenic) OR (persistent organic pollutants) OR (air pollution) OR (ambient temperature) OR (season) OR (air humidity)) |
| **Filters** | Language: English and German, until March 2021 | | | | |
| **Findings** | 64 | 409 | 0 | 34 | 312 |

**Figure 1: PRISMA flow chart**

Records identified through database searching

(n= 819)

Pubmed (n = 64)

Embase (n = 409)

Cochrane (n = 0)

CINHAL (n= 34)

Web of Science (n=312)

## Identification

Records after duplicates removed

(n = 515)

## Screening

Records excluded based on inclusion and exclusion criteria

(n = 410)

Records screened
(title and abstract)

(n =515)

## Eligibility

Full-text articles excluded

(n=14), reasons:

Wrong exposure (n = 3)

Wrong outcome (n = 5)

Retracted study (n = 1)

No full text (n = 5)

Full-text articles

assessed for eligibility

(n = 105)

Studies included in

qualitative synthesis

(n = 91)

## Included

Page MJ, McKenzie JE, Bossuyt PM, Boutron I, Hoffmann TC, Mulrow CD, et al. The PRISMA 2020 statement: an updated guideline for reporting systematic reviews. BMJ 2021;372:n71. doi: 10.1136/bmj.n71

**Table 2: Overview of included studies****: Bisphenol A**

| Author, Year | Location | Study design | Topic | Subjects (n, characteristics) | Main findings |
| --- | --- | --- | --- | --- | --- |
| Bellavia et al. 2018 | USA | Human study  Cross-sectional study | Pregnancy urinary **bisphenol-A** concentrations and glucose levels across BMI categories | - N= 350 pregnant women - Glucose levels were measured using a standard, non-fasting 50-g glucose load test (GLT) administered in the second trimester - Urinary BPA was measured in 1st and 2nd trimester in urine spot samples - measures were SG-adjusted and categorized into quartiles (Q) | - there was suggestive evidence of effect modification by maternal 1st trimester BMI, with **significant associations observed among obese/overweight participants** (1st trimester BPA concentrations for Q3 vs Q1: adj.β = 14.1 mg/dL; 95% CI: 1.5, 26.6) (p<0,05) - No associations were found between BPA and glucose levels in the overall population |
| Chiu et al. 2017 | USA | Human study  Prospective cohort study | Trimester-specific urinary **bisphenol a** concentrations and blood glucose levels among pregnant women from a fertility clinic | - n= 245 women (age 18-46) with a history of infertility - had available data on blood glucose levels from a 50-g glucose challenge test (GCT) - contributed at least one urine sample during first and/or second trimesters (208 samples in 1. trimester, 209 in 2.) - spot urine samples were collected during first and second trimesters of pregnancy | - Second-trimester BPA concentrations were positively associated with blood glucose (P, trend = 0.01) - the adjusted mean glucose levels (95% confidence interval) for women in the highest quartile of second-trimester BPA concentrations was 119 (112, 126) mg/dL compared with 106 (100, 112) mg/dL for women in the lowest quartile - **BPA exposure during the second trimester may have adverse effect on blood glucose levels among subfertile women** |
| Hou, Y. 2021 | China | Human study  Cross-sectional study | Associations of urinary **phenolic** environmental estrogens exposure with blood glucose levels and gestational diabetes mellitus in Chinese pregnant women | - n= 390 Chinese women at 24–28 weeks of gestation - GDM was diagnosed according to the diagnostic criteria of China's Ministry of Health and International Association of Diabetes and Pregnancy Study Groups recommendations - bisphenol A (BPA), nonylphenol (NP), and 2-tert-octylphenol (2-t-OP) concentrations were determined in urine samples - Linear and logistic regression tests evaluated associations of BPA, NP, and 2-t-OP with blood glucose levels and GDM prevalence | - Of the 390 pregnant women, 89 were diagnosed with GDM - significant positive associations between urinary 2-t-OP concentrations and FPG and 1-h PG levelswere observed when urinary BPA and NP were adjusted. - The 2-t-OP concentrations in GDM patients were significantly higher than in non-GDM women with median values of 2.23 μg/g Cr and 1.79 μg/g Cr, respectively. No significant difference was observed in BPA and NP. - Higher 2-t-OP levels were associated with higher odds of GDM (OR: 5.78; 95% CI: 2.04, 16.37), whereas higher NP levels were associated with lower odds (OR: 0.22; 95% CI: 0.05, 0.85) in the adjusted models. - compared to the first quartile of 2-t-OP, the adjusted odds ratios (ORs) and 95% confidence intervals (95% CIs) for GDM in the second, third, and fourth quartiles were 2.81 (1.23, 6.42), 3.01 (1.30, 6.93), and 5.49 (2.24, 13.46) - **exposure to 2-t-OP** **is associated with a higher risk of GDM (p<0,01). However,** **higher NP exposure is associated with lower GDM risk** |
| Robledo et al. 2013 | USA | Human study  Case-control-study | Is **bisphenol-A** exposure during pregnancy associated with blood glucose levels or diagnosis of gestational diabetes? | - N= 22 cases of GDM, Control: n=72 (older than 18) - total (free BPA + conjugates) urinary BPA concentrations (μg/L) were analysed in Urine specimens using a highperformance liquid chromatography (HPLC)– mass spectrometry (MS) method (online solidphase extraction [SPE] HPLC–MS/MS) with isotope dilution described previously - OGTT was used to diagnose GDM | - Logistic regression models controlling for race/ethnicity did not provide evidence of association between BPA exposure and case status across increasing tertiles of BPA exposure (number of GDM cases/controls in tertile1: 13/24; in tertile 2: 6/24; in tertile 3: 3/24). - **Findings do not support a relationship between total urinary BPA concentrations and altered glucose metabolism during pregnancy** |
| Wang et al. 2017 | China | Human study  Prospective Cohort-study | Urinary **bisphenol** concentration and gestational diabetes mellitus in Chinese women | - N= 620 pregnant women - From china 2012-2013 - During 24–28 weeks of gestation, all pregnant women were required routinely to undertake the 75-g OGTT - GDM was diagnosed according to the International Association of Diabetes and Pregnancy Study Groups - Urinary total BPA (free plus conjugated) concentration was quantified using a modified HPLC-MS/MS analytical method | - After adjustment for maternal age, education, husband smoking status, prepregnancy body mass index (BMI), and urinary creatinine concentration, plasma glucose at 2 hours in the 75-g OGTT was 0.36 mmol/L lower (95% confidence index [CI] = −0.73, 0.01) for women with urine BPA in the high versus the low tertile - For each unit increase in natural log-transformed BPA, the odds of GDM was reduced by 27% (odds ratio (OR) = 0.73; 95% CI = 0.56, 0.97), the birth weight decreased by 25.70 g (95% CI = −54.48, 3.07), and ponder index was decreased by 0.02 (100 g/cm3) (95% CI = −0.03, 0.00) - **Higher maternal urinary BPA concentrations were associated with reduced risk of GDM and marginally lower birth weight and ponder index** |
| Yang et al. 2021 | China | Human study  Prospective Cohort study | Serum **Bisphenol A**, glucose homeostasis, and gestational diabetes mellitus in Chinese pregnant women: a prospective study | - N= 535 pregnant women aged 20–40 years old in the early term of pregnancy (5–15 gestational weeks) - From china between 2013-2014 - Serum concentrations of BPA were measured in the early term of pregnancy using an isotope-dilution method based on ultraperformance liquid chromatography coupled with quadrupole time-of-flight mass spectrometry - fasting glucose and insulin levels were repeatedly measured in each of three terms of pregnancy - HOMAR-IR and OGTT were performed | - 33 women developed GDM - BPA was detected in 97.5% of pregnant women with a median of 6.50 ng/ml - Natural log-transformed BPA (Ln BPA) was positively associated with fasting glucose level (β (95% CI): 0.038 (0.015~0.061)), fasting insulin level (0.195 (0.069~0.321)), and homeostasis model insulin resistance index (HOMA-IR) (0.226 (0.087~0.364)) in the middle term of pregnancy (p<0,05) - Increased BPA concentration tended to increase the RR of GDM although not statistically significant (risk ratio: 2.51 (95% CI: 0.68~9.30) - **exposure to** **BPA might affect** **glucose homeostasis and the middle term of pregnancy was a potentially sensitive period.** |
| Zhang et al. 2019 | China | Human study  Prospective Cohort study | Exposure to **bisphenol A** substitutes and gestational diabetes mellitus: A prospective cohort study in China | - n= 1,841 pregnant women - from china between 2013-2015 - Concentrations of four bisphenols (BPA, BPS, BPF, BPAF) were measured in first-trimester urine samples - Urinary bisphenols concentrations were quantified using an Ultimate 3000 Ultra-high performance liquid chromatography system (Dionex, Sunnyvale, CA, USA) coupled to a Thermo ScientificTM TSQ QuantivaTM Triple Quadrupole mass spectrometer (Thermo Scientific, San Jose, CA) - Women undertake the OGTT | - Urinary BPAF was associated with increased odds of GDM among women with normal pre-pregnancy BMI [adjusted odds ratio (aOR) = 1.70 (95% CI: 1.08, 2.67) for the highest group compared to the lowest group], and the association remained significant after additional adjustment for other bisphenols [aOR = 1.68 (95% CI: 1.03, 2.72)] (p<0,01) - No significant associations were observed for other bisphenols and GDM. Consistent with the result of GDM, women in the highest BPAF category had a mean of 0.05 mmol/L (95% CI: 0.01, 0.09) higher fasting plasma glucose (FPG) levels than women in the lowest category. (p<0,01) - the per-unit increase in natural log transformed specific gravity adjusted BPS [ln (SG-adj BPS)] was associated with a 0.03 mmol/L (95% CI: 0.01, 0.04) increase in FPG levels and the associations might be modified by fetal sex (p for interaction < 0.05) - **results** **provide evidence that BPAF and** **BPS might be potential risk factors of GDM** |

**Table 3: Overview of included studies: Phthalate**

| Author, Year | Location | Study design | Topic | Subjects (n, characteristics) | Main findings |
| --- | --- | --- | --- | --- | --- |
| Chen et al. 2020 | China | Animal study  Intervention study | Maternal exposure to **Di-n-butyl phthalate (DBP**) aggravate gestational diabetes mellitus via FoxM1 suppression by pSTAT1 signalling | - N= 24 Sprague-Dawley rats - divided into 4 groups: STZ, DBP, DBP + STZ and Control group - changes in fasting blood glucose level in rats were detected on GD 1 and GD 5. The insulin levels in maternal rats and PIBCs were measured on GD 18 - the FoxM1 and pSTAT1 expression were measured in pancreas by immunohistochemistry | - Streptozotocin (STZ) + Di-n-butyl phthalate (DBP) group had higher glucose and lower insulin secretion levels than the other groups by both fasting test and OGTT - Forkhead box protein M1 (FoxM1) was significantly suppressed while Phosphorylated signal transducer and activator of transcription 1 (pSTAT1) was highly expressed after DBP exposure (p<0,05) - **DBP** **can influence the progression of GDM** through its toxicological effect, which significantly increases the expression of pSTAT1 and suppresses FoxM1, causing a decline in β cell viability (p<0,05) |
| Fisher et al. 2018 | UK | Human study  Prospective Cohort study | Serum **phthalate and triclosan** levels have opposing associations with risk factors for gestational diabetes mellitus | - n= 232 women aged ≤16 years without type 1 or 2 diabetes with singleton male pregnancies - Serum levels of 16 phthalate metabolites and 9 phenols (including BPA) were measured by isotope-diluted liquid chromatography coupled to tandem mass spectrometry - OGTT and insulin levels were measured | - First-trimester triclosan (TCS) was inversely associated with incident GDM (adjusted odds ratio per log increase in concentration 0.54, 95% confidence interval 0.34-0.86, p = 0.010). - Amongst women without GDM, first-trimester mono-(2-ethylhexyl) phthalate and mono(carboxyisooctyl) phthalate levels were positively associated with 120-min plasma glucose (adjusted β 0.268 and 0.183, p = 0.0002 and 0.010, respectively) in mid-pregnancy (p<0,01) - women with Mono-(2-ethylhexyl) phthalate (MEHP) levels in the second (aOR 25.82, 95% CI 1.79–372.52, p = 0.017) and fourth (aOR 23.22, 95% CI 1.68–320.03, p = 0.019) quartiles, bu not in the third quartile (aOR 8.09, 95% CI 0.59–111.44, p = 0.12), had significantly increased odds of GDM (phet = 0.08, ptrend = 0.041) - **The results support a glycaemia-raising effect of phthalates during pregnancy**, consistent with findings in non-pregnant populations and suggest a possible protective effect of exposure to TCS against GDM |
| Guo et al. 2021 | China | Human study  Cross-sectional-study | Meconium Exposure to **Phthalates**, Sex and Thyroid Hormones, Birth Size and Pregnancy Outcomes in 251 Mother-Infant Pairs from Shanghai | - n= 251 mother–infant pairs at the third trimester - 10 metabolites of phthalates in meconium samples collected during the first 24h after delivery were measured - Information about pregnancy outcomes (gestational diabetes, premature rupture of membrane, and premature birth) was available from the birth record. - GDM was diagnosed using a one-step approach with a 75g 2h-OGTT | - mono-iso-butylphthalate (MiBP), mono-n-butylphthalate (MnBP) and mono-2-ethyl-5-oxohexyl phthalate (MEOHP) were positively associated with birth length and femur length which seemed more obvious in female newborn; - MiBP, MnBP and mono-2-ethylhexylphthalate (MEHP) were significant positively associated with gestational diabetes mellitus (GDM) only in mothers with male newborns (p>0,05) - **This study indicates that meconium exposure to phthalates may adversely affect some fetal growth parameters and GDM with a potential gender effect** |
| James-Todd et al. 2018 | USA | Human study  prospective Cohort study | Environmental Health Trimester-specific **phthalate** concentrations and glucose levels among women from a fertility clinic | - N= 245 women 18 to 46 years of age who delivered live births - Spot urine samples collected prior to or at the time of the GCT test were used - phthalate concentrations were measured using solid phase extraction coupled with high performance liquid chromatographyisotope dilution tandem mass spectrometry | - Eighteen percent of women had glucose levels ≥ 140 mg/dL - Second trimester monoethyl phthalate (MEP) concentrations were positively associated with glucose levels, with adjusted mean (95%CI) glucose levels of 121 mg/dl (114, 128) vs. 109 mg/dL (103, 116) for women in highest and lowest quartiles (p<0,05) - Women in the highest quartile of second trimester mono-isobutyl phthalate (MiBP) concentrations had a mean glucose level 14 mg/dL lower compared to women in the lowest quartile. No other urinary phthalate metabolites were associated with glucose levels. - **MEP and MiBP—metabolites of diethyl phthalate and dibutyl phthalate, respectively—were associated with higher pregnancy glucose in subfertile women—a population at high risk of glucose intolerance in pregnancy** |
| James-Todd, Tamarra et al. 2016 | USA | Human study  Prospective Cohort study | Pregnancy urinary **phthalate** metabolite concentrations and gestational diabetes risk factors | - N= 350 women who delivered at term and had pregnancy urinary phthalate metabolite concentrations - Spot urine samples were collected at 9.9 week gestation (wg), 17.9 wg, 26.1 wg, 35.3 wg - Nine specific gravity-adjusted urinary phthalate metabolites were evaluated by National Science Foundation International, Inc. using protocol from the Centers for Disease Control and Prevention - First trimester BMI, GWG and Glucose levels in second and third trimester were measured (non-fasting 50-gram glucose load test (GLT) | - There were no associations between 1st trimester urinary phthalate metabolite concentrations and 1st trimester BMI - Mono-ethyl phthalate concentrations averaged across pregnancy were associated with 2.17 increased odds of excessive GWG (95% CI: 0.98, 4.79 - Second trimester mono-ethyl phthalate was associated with increased odds of impaired glucose tolerance (adj. OR: 7.18; 95% CI: 1.97, 26.15). - A summary measure of di-2-ethylhexyl phthalate metabolite concentrations were inversely associated with impaired glucose tolerance (adj. OR: 0.25; adj. 95% CI: 0.08, 0.85). - **Higher exposure to mono-ethyl phthalate, a metabolite of the parent compound of di-ethyl phthalate, may be associated with excessive GWG and impaired glucose tolerance** |
| Robledo et al. 2015 | UK | Human study  Cross-sectional-study | **Urinary phthalate** metabolite concentrations and blood glucose levels during pregnancy | - N= 72 pregnant women (18 years of age or older) who provided a spot urinary specimen - Blood glucose concentrations (mg/dl) were obtained by a one hour 50 g oral glucose challenge test - Urinary concentrations of nine phthalate metabolites and creatinine were measured in urine spot samples using online solid phase extraction coupled with highperformance liquid chromatography isotope dilution tandem massspectrometry as described elsewhere | - women with the highest urinary concentrations (≥3rd tertile) of mono-iso-butyl phthalate (tertile: ≥15.3 μg/l, β = −18.3, 95% CI: −35.4, −1.2) and monobenzyl phthalate (tertile: ≥30.3 μg/l, β = −17.3, 95% CI: −34.1, −0.4) had lower blood glucose levels at the time of GDM screening after adjustment for urinary creatinine and demographic covariates - Pregnant women with urinary concentrations of MiBP (β = −18.30 95% CI, −35.41 to −1.19) and MBzP (β = −17.26 95% CI, −34.12 to −0.40) in the highest concentration tertile had mean blood glucose levels approximately 18 mg/dl lower when compared to those in the 1st tertile after adjustment - findings showing inverse associations between urinary concentrations of MiBP and MBzP and blood glucose levels during pregnancy do not suggest that phthalate exposure is associated with insulin resistance and subsequently higher blood glucose levels. |
| Shaffer et al. 2019 | USA | Human study  Prospective Cohort study | Maternal **urinary phthalate** metabolites in relation to gestational diabetes and glucose intolerance during pregnancy | - N= 705 pregnant women (<13weeks pregnant, ≥18 years of age) - Data from T1 and third trimester (T3) urine samples were available for this analysis of 11 phthalates. T3 collection occurred concurrent with or after GDM screening. - two exposure metrics were utilized: 1) arithmetic mean of ln-transformed, SG-adjusted T1 and T3 urinary phthalate metabolite concentrations (“T1T3avg”), and 2) ln-transformed, SG-adjusted T1 urinary phthalate metabolite concentrations - GDM was diagnosed by GLT, and OGTT during wg 24-28 | - 60 cases of GDM, 90 cases of IGT, and an average GLT blood glucose of 113.6 ± 27.7 mg/dL were observed - average of T1 and third trimester (T1T3avg) monoethyl phthalate (MEP) was positively associated with GDM ([OR (95% CI) per IQR increase] T1T3avg MEP: 1.61 (1.10, 2.36)) - most other phthalates were not found to be related to study outcomes, though some associations were noted. - analyses suggested positive associations between phthalate metabolites and blood glucose difference for both exposure metrics but only the confidence intervals for MCOP excluded the null ((blood glucose difference (95%CI) per IQR increase) T1: 1.91 (0.25, 3.55), T1T3avg: 1.50 (0.02, 2.98)) - **an association between T1T3avg MEP and GDM was observed.** Additional phthalate metabolites were also found to be linked to glucose intolerance, with possible stronger associations in certain racial/ethnic subgroups |
| Shapiro et al. 2015 | Canada | Human study  longitudinal birth cohort study | Exposure to **phthalates, bisphenol A and metals** in pregnancy and the association with impaired glucose tolerance and gestational diabetes | - n= 1274 pregnant women (at least 18 years old) - between 2008 and 2011 - Phthalates in urine were analyzed by LC–MS/MS with an Ultra Performance Liquid Chromatography (UPLC) coupled with a tandem mass spectrometer and Quattro Premier XE following enzymatic deconjugation - IGT and GDM were assessed by GCT and OGTT | - Elevated odds of GDM were observed in the highest quartile of arsenic exposure (OR = 3.7, 95% CI = 1.4–9.6) in the adjusted analyses - A significant dose–response relationship was observed in a cubic spline model between arsenic and odds of GDM (p < 0.01) - No statistically significant associations were observed between phthalates or BPA or other metals with IGT or GDM - **The results are supporting the role of maternal arsenic exposure as a risk factor for gestational diabetes.** |
| Shapiro et al. 2018 | Canada | Human study  longitudinal birth cohort study | Associations between maternal **triclosan concentrations** in early pregnancy and gestational diabetes mellitus, impaired glucose tolerance, gestational weight gain and fetal markers of metabolic function | - N= 1795 pregnant women - between 2008 and 2011before 14 weeks gestation - associations of first-trimester urinary triclosan concentrations with total gestational weight gain, gestational diabetes mellitus and impaired glucose tolerance in pregnancy, and fetal markers of metabolic function - sensitive LC-MS/MS methods were developed for the analysis of free and conjugated forms of triclosan in urine - a 50 g GCT and a 75 or 100 g OGTT were conducted - Leptin and adiponectin were measured in plasma from umbilical cord blood samples, by ELISA using assay kits from Meso Scale Discovery (categorized - into<10th percentile, 10th–90th percentile, and>90th percentile) | - No significant associations were observed in adjusted analyses between triclosan concentrations and gestational diabetes mellitus, impaired glucose tolerance or gestational weight gain. - a non-significant inverse association between triclosan concentrations and leptin levels above the 90th percentile that was restricted to female fetuses (OR for highest quartile of triclosan compared to lowest quartile = 0.4 (95% CI 0.2–1.1), p-value for trend across quartiles = 0.02) was shown - Triclosan concentrations in the second quartile were associated with elevated odds of adiponectin below the 10th percentile in male fetuses (OR for Q2 compared to Q1 = 2.5, 95% CI 1.1–5.9, p-value for trend across quartiles = 0.93). - **This study does not support an association between triclosan concentrations in pregnancy and fetal metabolic markers,** **glucose disorders of pregnancy, or excessive gestational weight gain** |

**Table 4: Overview of included studies: POPs**

| Author, Year | Location | Study design | Topic | Subjects (n, characteristics) | Main findings |
| --- | --- | --- | --- | --- | --- |
| Alvarez-Silvares et al. 2021 | Spain | Human study  Case-control study | Association between placenta concentrations **polybrominated and polychlorinated biphenyls** and gestational diabetes mellitus: a case-control study in north-western Spain | - N = 86 pregnant women - Case group: women who developed GDM during the course of their current pregnancy, diagnosed by means of OGTT - Control group: women who did not develop GDM - Selective pressurized liquid extraction methodologies were used to determine targeted persistent organic pollutants in placenta samples | - concentrations of several congeners of polybrominated diphenyl ethers (PBDE) and polychlorinated biphenyls (PCB) presented significant lower levels in the placentas of expectant mothers with gestational diabetes mellitus (p<0,05) - this association was independent of obesity, age, parity, amenorrhoea at birth, or a family history of diabetes mellitus. - **an inverse relationship between PBDE and PCB levels in placenta and gestational diabetes mellitus was reported** |
| Bellavia et al. 2019 | USA | Human study  Prospective Cohort study | Urinary concentrations of **parabens mixture** and pregnancy glucose levels among women from a fertility clinic | - N= 241 pregnant women - Between 2005 and 2015 - blood glucose levels assessed through a 1-hour non-fasting, 50-gram GLT - urinary methylparaben, propylparaben, and butylparaben concentrations, and blood glucose levels were measured in up to two spot urine samples in 1st and/or 2nd trimester - Trimester-specific associations between specific gravity adjusted methylparaben, butylparaben, and propylparaben with adjusted mean of pregnancy glucose levels were evaluated | - positive associations of butylparaben (e.g comparing the 4th and 1st quartiles) with glucose levels, for both the 1st trimester (adjusted difference=12.5 mg/dL; 95% CI: 0.9, 24.2) and 2nd trimester (adjusted difference=11.2 mg/dL; 95% CI: 0.2, 22.3) (p<0,05) - 1st trimester butylparaben and propylparaben urinary concentrations are associated with glucose levels in a pregnancy cohort of women at high risk of GDM - **Parabens may be involved in the development of GDM a**nd should be further evaluated. |
| Eslami et al. 2016 | Iran | Human study  Case-control-study | Association between serum concentrations of **persistent organic pollutants** and gestational diabetes mellitus in primiparous women | - N= 70 Serum samples from cases diagnosed with GDM - Control: n=70 - polychlorinated biphenyls (PCBs, 10 congeners) and polybrominated diphenyl ethers (PBDEs, 8 congeners) were analysed in 10ml blood samples - Diagnosis of GDM was based on the International Association of Diabetes and Pregnancy Study Groups (IADPSG) criteria | - the positive association between total POPs (sum of total PCBs and PBDEs) (Odds ratio (OR)=1.61, 95% CI: 1.31-1.97, p-value <0.0001) and total PCBs (OR=1.75, 95% CI: 1.35-2.27, p-value<0.0001) and GDM was manifested - a positive association between total PBDEs and GDM (OR =2.21; 95% CI: 1.48-3.30, p-value <0.0001) was found - a positive association between Ln PCB 187, 118 and Ln PBDE 99, 28 with GDM was found (p<0,05) - **data suggest that exposure to certain POPs (PCBs and PBDEs) could be a potential modifying risk factor for GDM** |
| Jaacks et al. 2016 | USA | Human study  Prospective Cohort study | Pre-pregnancy maternal exposure to **polybrominated and polychlorinated biphenyls** and gestational diabetes: a prospective cohort study | - n= 258 women (aged 18–40) who achieved pregnancy lasting ≥24 weeks gestation and completed monthly pregnancy journal - between 2005 – 2007 - association between maternal pre-pregnancy levels of a polybrominated biphenyl (PBB 153) and 36 polychlorinated biphenyls (PCBs) with gestational diabetes (GDM) was estimated - Women who ever reported a physician diagnosis of high blood glucose during pregnancy that was not pre-existing were categorized as having gestational diabetes (n = 28; 10.9 %). | - There was no association between PBB 153 and GDM or any of the PCB congeners and GDM in unadjusted models - All associations remained non-significant with stepwise adjustment for age and waist-to-height ratio - with further adjustment for total serum lipids did the associations become significant, with lower levels of nine PCB congeners associated with GDM: 138, 153, 156, 167, 170, 172, 178, 180, and 194. The adjusted ORs for PCBs 170 and 180 were the strongest: 0.40 (0.18, 0.88) and 0.41 (0.19, 0.87) (p<0,05) - **Pre-pregnancy levels of PCBs were not consistently associated with development of GDM** |
| Liu et al. 2019 | China | Human study  Prospective Cohort study | **Parabens** exposure in early pregnancy and gestational diabetes mellitus | - N= 1087 pregnant women (mean age: 26.4) - Between 2014 and 2015 in Wuhan - Parabens [methyl paraben (MeP), ethylparaben (EtP), propylparaben (PrP), butylparaben (BuP), and benzylparaben (BzP)] concentrations were measured in spot urine samples collected between 8 and 16 gestational weeks - GDM was assessed by 75 g OGTT | - A total of 103 (9.5%) women were diagnosed with GDM. - After adjustment for potential confounders, urinary EtP was associated with GDM. The risk ratios (RRs) = 1.12 (95% CI: 0.63, 2.01) for the second quartile, RRs = 1.11 (95% CI: 0.64, 1.93) for the third quartile, and RRs = 1.70 (95% CI: 1.02, 2.82) for the highest quartile, compared with the lowest quartile (p<0,05) - There was no evidence of associations between urinary MeP or PrP and GDM - **Results suggest that exposure to EtP may increase the risk of GDM.** |
| Liu et al. 2019 | China | Human study  case-control study | Structure-based investigation on the association between **perfluoroalkyl acids exposure** and both gestational diabetes mellitus and glucose homeostasis in pregnant | - N (cases): 77 pregnant women who developed GDM at 24–28 weeks of gestation - N (control) = 126 - Between 2013–2015 in Beijing - First trimester maternal serum was collected and analysed for 25 diverse PFAAs using standard laboratory quality assurance (QA) and quality control (QC) protocols - GDM was diagnosed according to a “One-step” approach using a OGTT | - 12 perfluoroalkyl carboxylates (PFCAs) and 8 perfluoroalkyl sulfonates (PFSAs) were detected in >55.0% of samples and were respectively grouped into different structural groups - The structural-based effect was observed for PFCAs, where short-chain (C4-C7) PFCAs continuous level was significantly associated with GDM with an estimated odds ratio (OR) of 1.99 (95% CI: 1.29, 3.09), and the multivariable-adjusted ORs (95% CI) of GDM for increasing tertiles of short-chain PFCAs were 1.00 (ref.), 1.82 (0.80, 4.16) and 3.01 (1.31, 6.94), P trend = 0.011 - increased concentration of short-chain PFCAs was significantly associated with higher postprandial glucose levels (P < 0.05). - **the investigation suggests a structure-specific association between short-chain PFCAs exposure and both GDM risk and impaired glucose homeostasis in pregnant women** |
| Liu et al. 2018 | China | Human study  Nested case-control study | A nested case-control study of the association between exposure to **polybrominated diphenyl ethers** and the risk of gestational diabetes | - n= 77 GDM cases - n= 154 controls - between 2013 and 2015 - diagnosis of GDM was made following the Diagnostic Criteria - for Gestational Diabetes Mellitus released by the Ministry of Health of China - Seven predominant PBDE congeners were measured in first trimester maternal serum using standard laboratory QA/QC protocols | - A total of 77 (17.5%) women developed GDM in this study - significant associations between BDE-153, -154, -183 and GDM risk with an estimated odds ratio of 4.04 (95%CI: 1.92, 8.52), 1.88 (95%CI: 1.15, 3.09) and 1.91 (95%CI: 1.31, 2.08) is suggested - a significant increase in the odds ratio of GDM was associated with the highest levels of BDE-153 (OR = 3.42 95%CI: 1.49, 7.89) and BDE-183 (OR = 3.70, 95%CI: 1.58, 8.65), whereas, BDE-154 demonstrated an inverted U-shaped association with GDM - BDE-153 and -154 were significantly positively associated with fasting glucose, and both 1 h and 2 h glucose level (p < 0.05) - **exposure to PBDEs** **disturbs maternal glucose homeostasis and increases the risk of GDM** |
| Rahman et al. 2019 | USA | Human study  Prospective cohort study | **Persistent organic pollutants** and gestational diabetes: A multi-center prospective cohort study of healthy US women | - n= 2334 healthy non-obese women at 8–13 weeks of gestation - between 2009 - 2013 - plasma concentration of 76 POPs, including 11 organochlorine pesticides (OCPs), 9 polybrominated diphenylethers (PBDEs), 44 polychlorinated biphenyls (PCBs), and 11 per-and polyfluoroalkyl substances (PFAS) were measured - All women underwent a 100 g 3-h OGTT test except for 123 participants who underwent a 75 g 2-h OGTT test. | - Higher concentrations of PCBs with six or more chlorine atoms were associated with increased risk of GDM in the overall cohort (risk ratios [RRs] range: 1.08–1.13 per 1-standard deviation [SD] increment) and among women with a family history of T2D (RRs range: 1.08–1.48 per 1-SD increment) or normal ppBMI (RRs range: 1.08–1.22 per 1-SD increment) - four PFAS congeners - perfluorononanoic acid (PFNA), perfluorooctanoic acid (PFOA), perfluoroheptanoic acid (PFHpA), and perfluorododecanoic acid (PFDoDA) - showed significant positive associations with GDM among women with a family history of T2D (RRs range:1.22–3.18 per 1-SD increment) - BDE47 and BDE153 showed significant positive associations among women without a family history of T2D (p<0,05) - **Environmentally relevant levels of heavily chlorinated PCBs and some PFAS and PBDEs were positively associated with GDM with suggestive effect modifications by family history of T2D and body adiposity status** |
| Saunders et al 2014 | Guadeloupe | Human study  prospective epidemiological mother–child cohort study | Hypertensive disorders of pregnancy and gestational diabetes mellitus among French Caribbean women chronically exposed to **chlordecone** | - N= 779 pregnant women - between 2004 and 2007 - maternal plasma was assayed for chlordecone - Samples from a subgroup of 358 women were also assayed for DDE and PCB 153 - impact of prenatal exposure to chlordecone on the occurrence of GDM, gestational hypertension (GH) and preeclampsia (PE) was investigated - Pregnant women were systematically screened for GDM using between 24 and 28 weeks of gestation using GCT and OGTT | - Levels of chlordecone plasma concentration in the third (OR = 0.2; 95% confidence interval (CI): 0.1, 0.5) and fourth quartiles (OR = 0.3; 95% CI: 0.2, 0.7) were associated with a statistically significant decrease in the risk of GH - **No significant associations were observed between the chlordecone exposure and the risk of PE or GDM** - This study suggests an inverse association between chlordecone exposure during pregnancy and GH (p<0,05) |
| Shapiro et al. 2016 | Canada | Human study  Longitudinal birth cohort study | Exposure **to organophosphorus and organochlorine pesticides, perfluoroalkyl substances, and polychlorinated biphenyls** in pregnancy and the association with impaired glucose tolerance and gestational diabetes mellitus | - N= 1274 women - Exposure variables included three organophosphorus (OP) pesticide metabolites detected in first-trimester urine samples, as well as three organochlorine (OC) pesticides, three PFASs, and four PCBs in first-trimester blood samples - GDM was diagnosed by GCT and/or OGTT | - Significantly lower odds of GDM were observed in the third and fourth quartiles of dimethylphosphate (DMP) and in the fourth quartile of dimethylthiophosphate (DMTP) in adjusted analyses (DMP Q3: OR=0.2, 95% CI=0.1–0.7; DMP Q4: OR=0.3, 95% CI=0.1–0.8; DMTP: OR=0.3, 95% CI=0.1–0.9) - Significantly elevated odds of gestational IGT was observed in the second quartile of perfluorohexane sulfonate (PFHxS) (OR=3.5, 95% CI=1.4–8.9) (p<0,05) - No evidence of associations with GDM or IGT during pregnancy was observed or PCBs or OC pesticides. - **There was no consistent evidence for any positive associations between the chemicals we examined and GDM or IGT during pregnancy**. We observed statistical evidence of inverse relationships between urine concentrations of DMP and DMTP with GDM |
| Smarr et al. 2016 | USA | Human study  Longitudinal Cohort study | **Persistent organic pollutants** and pregnancy complications | - N= 258 women - Between 2005 and 2009 - Preconception concentrations of 9 organochlorine pesticides (OCPs) and 10 polybrominated diphenyl ethers (PBDEs) were quantified in serum - GDM was defined as physician report high blood sugar - gestational hypertension was a second outcome | - Women's serum concentration of PBDE congener 153 (PBDE-153) was positively associated with an increased odd of GDM per SD increase in log-transformed concentration, for unadjusted (OR = 1.36, 95%CI: 1.02–1.81), a priori adjusted (OR = 1.38, 95% CI: 1.03–1.86) and with the sum of remaining PBDEs (OR = 1.79, 95% CI: 1.18, 2.74) models (p<0,05) - **at environmentally relevant concentrations, maternal exposure to POPs prior to conception may contribute to increased chance of developing GDM**. |
| Vafeiadi et al. 2017 | Greece | Human study  population-based Cohort study | **Persistent organic pollutants** in early pregnancy and risk of gestational diabetes mellitus | - n= 939 pregnant women - Concentrations of several PCBs, dichlorodiphenyldichloroethene (DDE), and hexachlorobenzene (HCB) were determined in first trimester maternal serum using an Agilent 7000B gas chromatograph triple quadrupolemass spectrometer (GC-MS/MS) - Women were screened for GDM at 24–28 week | - 68 (7%) women developed GDM - Women in the medium and high tertiles of PCBs had 3.90 (95% CI: 1.37, 11.06) and 3.60 (95% CI: 1.14, 11.39) fold respectively higher odds of developing GDM compared to women in the lowest tertile of PCB exposure (p<0,001) - Odds of GDM for women in the medium and high tertiles of dioxin-like PCBs was 5.63 (95% CI: 1.81, 17.51) and 4.71 (95% CI: 1.38, 16.01) and for nondioxin-like PCBs 2.36 (95% CI: 0.89, 6.23) and 2.26 (95% CI: 0.77, 6.68) - Prenatal DDE and HCB exposure were not significantly associated GDM risk. - **women with high PCBs levels in early pregnancy had higher risk for GDM** |
| Valvi et al. 2017 | USA | Human study  Prospective Cohort study | Gestational diabetes and offspring birth size at elevated **environmental pollutant** exposures | - n= 604 Faroese pregnant women and their offspring - Maternal pregnancy serum concentrations of organochlorine compounds (OCs: polychlorinated biphenyl (PCB) congeners and dichlorodiphenyldichloroethylene (DDE)), and five perfluoroalkyl substances (PFASs), and hair and cord blood mercury concentrations were measured - GDM diagnosis was extracted from the medical records and women with elevated risk undertook a OGTT | - Serum-DDE and hair-mercury concentrations were associated with GDM (adjusted OR per concentration doubling: 1.29; 95% CI: 0.94, 1.77 for DDE, and 0.79; 95% CI: 0.62, 0.99 for mercury) but in multiple pollutant-adjusted SEMs only a positive association between OC exposure and GDM remained significant (change in GDM odds per OC doubling: 0.45; 95% CI: 0.05, 0.86) (p<0,05) - PCB and overall OC exposure were positively associated with head circumference (SEM; mean change per OC doubling: 0.13 cm; 95% CI, 0.01. 0.25) - Overall PFAS exposure was inversely associated with birth weight (SEM; mean change per PFAS doubling: − 169 g; 95% CI: − 359, 21), and for many single-PFASs we found a pattern of inverse associations with birth weight and head circumference in boys, and positive or null associations in girls - **associations with GDM and offspring birth size were found to be specific to the environmental pollutant or pollutant group. Associations with birth size measures appear to be independent of GDM occurrence.** |
| Wang et al. 2018 | China | Human study  Case-control-study | Association of serum levels of **perfluoroalkyl substances** with gestational diabetes mellitus and postpartum blood glucose | - n= 84 women with GDM - n= 168 healthy pregnant women - maternal blood was collected at 1–2 days before delivery - eight linear isomers and fourteen branched isomers were determined in maternal serum - diagnoses of GDM follow the Diagnostic Criteria for Gestational Diabetes Mellitus released by Ministry of Health of China | - Although maternal PFAS exposure was not associated with risk of GDM, significant positive associations were observed between evaluated exposure to specific PFAS congeners and increasing blood glucose. (p<0,05) - The odds ratio (ORs) of the highest category of postpartum fasting blood glucose for perfluoro-1-metylheptylsulfonat (1m-PFOS), perfluoro-3/4-metylheptylsulfonat (3m + 4m-PFOS), perfluoro-5-metylheptylsulfonat (5m-PFOS), and perfluorohexane sulfonate (PFHxS) were 2.03 (95% CI: 1.09–3.77), 1.93 (95% CI: 1.04–3.58), 2.48 (95% CI: 1.33–4.65), and 2.26 (95% CI: 1.21–4.21), respectively, **suggesting negative effects of maternal exposure to specific PFAS compounds on glucose metabolism** |
| Wang et al. 2020 | USA | Human study  Prospective Cohort study | Perinatal urinary **benzophenone-3** concentrations and glucose levels among women from a fertility clinic | - N= 217 pregnant women - from 2009 to 2017 - women who had urinary BP-3 concentrations measured during 3-month preconception (178 samples), first (194) and/or second trimesters (170) in spot urine samples - blood glucose measured at glucose load tests (GLTs) during late pregnancy | - Women with higher first trimester BP-3 concentrations had lower mean glucose levels [mean glucose (95% CI) for Q4 vs Q1 = 103.4 (95.0, 112.5) vs. 114.6 (105.8, 124.2) mg/dL] - Women with higher second trimester BP-3 concentrations had lower odds of abnormal GLT [OR (95% CI) for Q3 vs. Q1 = 0.12 (0.01, 0.94)]. - women with female-factor infertility, urine collected during summer, older age, lower BMI, or carried female fetus (es) had the strongest inverse associations between BP-3 and glucose levels, while no associations were observed in the remaining subgroups (p<0,05) - **Time-specific inverse associations between BP-3 and pregnancy glucose levels existed in subfertile women, and especially among certain subgroups of this high-risk-population** |
| Xu et al. 2020 | China | Human study  Case-control-study | Exposure to elevated **per- and polyfluoroalkyl substances** in early pregnancy is related to increased risk of gestational diabetes mellitus: A nested case-control study in Shanghai, China | - N= 165 GDM cases - N= 330 controls - Between 2017 - 2019 - Twelve PFASs of interest were measured in the sera of pregnant women at 16–20 weeks - Screening for GDM was examined over 24–28 gestational weeks using OGTT | - The serum levels of perfluorobutanesulfonic acid (PFBS) and perfluorododecanoic acid (PFDoA) were significantly higher in the GDM group than they were in the control group (P = 0.02 and P < 0.01, respectively) - A quartile analysis showed that the odds ratio of GDM would significantly increase at the highest PFBS and PFDoA levels - In the core model, the adjusted ORs were 2.02 (95% CI = 1.04–3.79) and 13.00 (95% CI = 4.74–24.59), respectively, after adjusting for maternal age, sampling time, parity and body mass index [BMI]) - **Elevated maternal serum PFBS and PFDoA levels in early pregnancy may be associated with a substantially higher GDM risk** |
| Zhang et al. 2018 | China | Human study  Nested Case-control-study | **Non-dioxin-like polychlorinated biphenyls** in early pregnancy and risk of gestational diabetes mellitus | - N= 77 women with GDM - N= 154 control - Participants were screened for GDM at 24–28 weeks of gestation following the Diagnostic Criteria for Gestational Diabetes Mellitus - Concentrations of selected so-called ‘indicator PCBs’ were determined in sera of women in the first trimester of pregnancy | - The odds ratios (OR) of PCB-28, PCB-52, and PCB-101 for GDM were 1.86 (95% CI: 1.05–3.27), 1.90 (95% CI: 1.28–2.82) and 1.85 (95% CI: 1.22–2.82), respectively - after adjusting for confounders including some PCB congeners, only PCB-52 remained significantly associated with GDM with OR of 1.97 (95% CI: 1.27–3.07). - PCB-52 was positively associated with all blood glucose values of OGTT (p < 0.05). - **serum levels of specific non-dioxin-like PCBs in early pregnancy disturb the glucose metabolism and increase the risk of GDM.** |

**Table 5: Overview of included studies: Arsenic**

| Author, Year | Location | Study design | Topic | Subjects (n, characteristics) | Main findings |
| --- | --- | --- | --- | --- | --- |
| Ashley-Martin et al. 2018 | USA | Human study  Prospective Cohort study | Association between maternal urinary speciated **arsenic** concentrations and gestational diabetes in a cohort of Canadian women | - n = 1243 women (>18 years), with complete urinary arsenic and glucose testing data - between 2008 and 2011 - Concentrations of speciated As, methylated arsenic species metabolites and organic were measured in first trimester maternal urine samples - IGT and GDM were diagnosed using guidelines from the Canadian Diabetes Association and the Society of Obstetricians and Gynaecologists of Canada | - Compared to women in the lowest tertile of dimethylarsinic acid (DMA) (<1.49 μg As/L), women with concentrations exceeding 3.52 μg As/L (3rd tertile) experienced an increased risk of GDM (aOR = 3.86; 95% CI: 1.18, 12.57) (p-value for trend across tertiles = 0.04). - When restricted to women carrying male infants, the magnitude of this association increased (aOR 3rd tertile = 4.71; 95% CI: 1.05, 21.10) - **These results suggest** **a positive relation between DMA and GDM** |
| Bonaventura et al. 2017 | Argentina | Animal study  Intervention study | **Arsenite** in drinking water produces glucose intolerance in pregnant rats and their female offspring | - Pregnant A50 rats and their offspring - 5 (A5) or 50 (A50) mg/L of sodium arsenite in drinking water was administered from gestational day 1 (GD1) until two months postpartum (2MPP), and to their offspring from weaning until 8 weeks old - glucose tolerance test, insulin secretion test and HOMA of insulin resistance was evaluated on GD16-17 and litter parameters at birth - arsenic content in liver samples from dams sacrificed at two months postpartum (2MPP), and from offspring at two months of age was determined | - Arsenite-treated pregnant animals showed glucose intolerance on GD16-17, with impaired insulin secretion but normal insulin sensitivity; they showed dose-dependent increased pancreas insulin on GD18 (p<0,05) - All alterations reverted at 2MPP - Offspring from A50-treated mothers showed lower body weight at birth, 4 and 8 weeks of age, and glucose intolerance in adult females, probably due to insulin secretion and sensitivity alterations (p<0,05) - **Arsenic** **alters glucose homeostasis during pregnancy by altering beta-cell function, increasing risk of developing gestational diabetes** |
| Ettinger et al. 2009 | USA | Human study  Prospective Cohort study | Maternal **arsenic** exposure and impaired glucose tolerance during pregnancy | - n=532 women - 2002-2008 - it was investigated if arsenic exposure is associated with impaired glucose tolerance during pregnancy - Blood and hair were collected at delivery and analysed for arsenic - Blood glucose was measured at a prenatal visit between 24 and 28 weeks gestation after a 1-hr, 50-g oral glucose tolerance test (GTT) | - Arsenic concentrations ranged from 0.2 to 24.1 microg/L (ppb) (mean +/- SD, 1.7 +/-1.5) and 1.1 to 724.4 ng/g (ppb) (mean +/- SD, 27.4 +/- 61.6) in blood and hair, respectively - impaired glucose tolerance was observed in 11.9% of women when using standard screening criterion (> 140 mg/dL) - women in the highest quartile of blood arsenic exposure had 2.8 higher odds of impaired GTT than women in the lowest quartile of exposure (95% confidence interval, 1.1-6.9) (p-trend = 0.008) - **arsenic exposure was associated with increased risk of impaired GTT at 24-28 weeks gestation and therefore may be associated with increased risk of GD.** |
| Farzan et al. 2016 | USA | Human study  Prospective Cohort study | Maternal **arsenic** exposure and gestational diabetes and glucose intolerance in the New Hampshire birth cohort study | - n= 1151 pregnant women at 24-28 weeks gestation - association between As exposure and risk of glucose intolerance and GDM was investigated - Arsenic was measured in home well water and via biomarkers - GDM was diagnosed by GCT and/or OGTT | - A total of 105 (9.1 %) of women were diagnosed with glucose intolerance and 14 (1.2 %) of women were diagnosed with GDM - As concentration in home well water was associated with a ~10 % increased odds of GDM (OR: 1.1, 95 % CI 1.0, 1.2). - A positive and statistically significant association also was observed between toenail As and GDM (OR: 4.5, 95 % CI 1.2, 16.6), but not urinary arsenic (OR: 0.8, 95 % CI 0.3, 2.4) (p<0,05) - the association between water As and GDM and glucose intolerance was largely limited to obese women (OR: 1.7, 95 % CI 1.0, 2.8). - There was no evidence of an association for As exposure with glucose intolerance - **the role of As exposure via water from private wells in the incidence of GDM and that this association may be modified by body composition is supported** |
| Marie et al. 2018 | France | Human study  semi-ecological study | Exposure to **arsenic** in tap water and gestational diabetes: A French semi-ecological study | - n= 5053 women - from 2003 – 2006 and in 2010 - screening of GDM was performed between 24 and 28 weeks of pregnancy with GCT and OGTT - As exposure was estimated from the concentrations of As measured during sanitary control of tap water supplied in the mothers’ commune of residence | - Overall, women in the As + group had a higher risk of GDM than those in the As - group (adjusted OR = 1.62; 95%CI: 1.01–2.53) (p<0,05) - Stratified analysis of pre-pregnancy body mass index (BMI) showed a positive association only for obese or overweight women (adjusted OR = 2.30; 95%CI: 1.13–4.50) (p<0,05) - **The results provide additional arguments for an association between As exposure and the risk of GDM in particular in a context of low exposure** |
| Muñoz et al. 2018 | Chile | Human study  Cross-sectional-study | Urinary Inorganic **Arsenic** Concentration and Gestational Diabetes Mellitus in Pregnant Women from Arica, Chile | - N= 244 pregnant women - association between urinary inorganic arsenic concentration and gestational diabetes among pregnant women was evaluated - GDM was diagnosed using the diagnostic criteria of the World Health Organization (WHO) for diabetes | - median urinary inorganic arsenic was 14.95 &mu;g/L, and the prevalence of gestational diabetes was 8.6% - there was no significant association between gestational diabetes and inorganic arsenic exposure tertiles (Odds ratio (OR) 2.98, 95% CI = 0.87⁻10.18), (OR 1.07, 95% CI = 0.26⁻4.33). - **This study** **did not provide evidence on the relationship between urinary inorganic arsenic concentration and gestational diabetes.** |
| Peng et al. 2015 | China | Human study  Case-control-study | A nested case-control study indicating **heavy metal** residues in meconium associate with maternal gestational diabetes mellitus risk | - N= 137 pregnant women with GDM - N= 190 healthy pregnant women = control - Arsenic (As), mercury (Hg), lead (Pb), cadmium (Cd), and chromium (Cr) levels in the case-control meconium samples were measured - GDM was diagnosed using OCT and OGTT | - The concentrations of As, Hg, Cr and Cd in studied cases were significantly higher (p < 0.05) than those of controls - As, Cd and Cr were found to be positively associated with GDM prevalence in dose-dependent manners (p<0,001) - As was detected in all samples and its levels associated the maternal GDM with the adjusted odds ratios of 3.28 [95% CI 1.24, 8.71], 3.35 [95% CI 1.28, 8.75] and 5.25 [95% CI 1.99, 13.86] for the 2(nd), 3(rd) and 4(th) quartiles, respectively - **exposure to some of the selected metals (noticeably As) may contribute to maternal GDM risk during pregnancy** |
| Wang et al. 2020 | China | Human study  Prospective Cohort study | Exposure to **multiple metals** in early pregnancy and gestational diabetes mellitus: A prospective cohort study | - n= 2090 women with singleton pregnancy - spot urine samples were collected before 20 gestational weeks - concentrations of urinary metals were measured - GDM was diagnosed using OGTT between 24 and 28 gestational weeks | - Five metals (Ni, As, Sb, Co, and V) were found significantly and positively associated with GDM based on single-metal models (p<0,05) - In multiple-metal models, for each unit increase of ln-transformed urinary Ni or Sb, the risk of GDM increased 18% [relative risk (RR):1.18, 95%confidence interval (CI): 1.00, 1.38 or RR: 1.18, 95%CI: 1.00, 1.39, respectively]. - The effect of metal Ni was significant (p<0,1) when the concentrations of the other metals were all fixed at their 25th percentile, and metal Sb displayed a significant and positive effect when all the other metals were fixed at 25th, 50th, and 75th percentiles - **All six metals mixed** **exposure was positively associated with the risk of GDM, while Sb and Ni were demonstrated more important effects than the other four metals in the mixture** |
| Wang et al. 2019 | China | Human study  Case-control-study | **Multiple metal** concentrations and gestational diabetes mellitus in Taiyuan, China | - n= 776 women with GDM - n= 776 controls - GDM diagnosis was based on a 75-g oral glucose tolerance test (OGTT) during gestational weeks 24 and 28 - Concentrations of metals in participants’ blood (nickel (Ni), arsenic (As), cadmium (Cd), antimony (Sb), thallium (Tl), mercury (Hg), lead (Pb)) were measured using inductively coupled plasma-mass | - An increased risk of GDM was associated with As (OR = 1.49, 95% CI: 1.11, 2.01 for the 2nd tertile vs. the 1st tertile) and Hg (OR = 1.43, 95% CI: 1.09, 1.88 for the 3rd tertile vs. the 1st tertile) - the weighted quantile sum (WQS) index was significantly associated with GDM (OR = 1.20, 95% CI: 1.02, 1.41). - **The major contributor to the metal mixture index was Hg (69.2%), followed by Pb (12.8%), and As (11.3%)** - the second principal component, which was characterized by Hg, Ni, and Pb, was associated with an increased risk of GDM (OR = 1.46, 95% CI: 1.02, 2.08 for the highest quartile vs. the lowest quartile). |
| Wu et al. 2018 | China | Human study  Case-control-study | **Multiple elements** related to metabolic markers in the context of gestational diabetes mellitus | - n = 137 GDM cases - n = 197 controls - Twenty-one meconium elements were characterized - diagnosis of GDM was retrieved according to the World Health Organization's criteria | - Eight elements were related with the GDM occurrence in dose-dependent manners, which positively (Al, As, Ba, Cd, Hg, and Sn) or negatively (Ca and V) associated with GDM - As, Cd, Ba, and Ca significantly contributed to the variation of GDM-related metabolic markers (p<0,05) - the associations of Cd, Ba, Ca and As with GDM were mediated by the metabolic markers which majorly involved in the lipid metabolism and the Adenosine/l-Arginine/Nitric Oxide (ALANO) pathways - **maternal exposure to As, Ba, Cd, and Ca may be associated with the dysfunction of fetus development niche through disrupting lipid metabolism and ALANO pathways** |
| Xia et al. 2018 | China | Humasn study  Prospective Cohort study | Association between serum **arsenic** levels and gestational diabetes mellitus: A population-based birth cohort study | - n= 3260 pregnant women - between 2013 and 2014 - GDM was diagnosed based on the criteria from American Diabetes Association - Serum As concentrations were determined at multiple time points during pregnancy by ICP-MS using our previously established method - The association between As levels and GDM prevalence was examined | - A total of 419 (12.85%) women were diagnosed with GDM - The incidences of GDM gradually increased with increasing quartiles of As levels with significant trend - As levels were associated with the GDM (95%CI: 1.29–2.43) at only the 4th quartile in the first trimester (p<0,05) - **The results showed an association between As and GDM in a birth cohort and explored first trimester may be the critical period for As associated GDM** |

**Table 6: Overview of included studies: Cadmium**

| Author, Year | Location | Study design | Topic | Subjects (n, characteristics) | Main findings |
| --- | --- | --- | --- | --- | --- |
| Li et al. 2020 | China | Human study  Case-control-study | Association of **urinary cadmium**, circulating fatty acids, and risk of gestational diabetes mellitus: A nested case-control study in China | - N= 305 GDM cases - N= 305 controls - Urinary cadmium (Cd) concentrations and levels of plasma fatty acids (FAs) between 10 and 16 gestational weeks were measured in spot urine samples at 13 weeks gestation - diagnosis of GDM was based on the recommended criterion by International Association of Diabetes and Pregnancy Study Group | - Urinary concentrations of Cd in cases (median: 0.69 μg/L) were significantly higher than controls (median: 0.59 μg/L, P < 0.05) - Cd concentrations were positively associated with the risk of GDM (Ptrend = 0.003) - Cd concentrations were positively correlated with levels of eicosadienoic acid and arachidonic acid/eicosapentaenoic acid ratio, but negatively correlated with levels of stearic acid, eicosapentaenoic acid, total odd-chain saturated fatty acids, total n-3 polyunsaturated fatty acids (PUFAs), and n-3 PUFAs/n-6 PUFAs ratio - **The findings confirmed the association of higher Cd exposure with increased risk of GDM in pregnant women,** and provided forceful epidemiological evidence for the relation of Cd concentrations and levels of FAs |
| Liu et al. 2018 | China | Human study  Prospective Cohort study | **Cadmium** Body Burden and Gestational Diabetes Mellitus: A Prospective Study | - N= 2,026 pregnant women (over 16 wg) - between 2013 and 2016 in Wuhan - Cd body burden was reflected by Cd concentrations in spot urine samples collected between gestational weeks 8 and 14 - Women were diagnosed with GDM according to International Association of Diabetes and Pregnancy Study Group | - A total of 198 (9.8%) women were diagnosed with GDM - the risk ratios (RRs) of GDM were 1.04 (95% CI: 0.74, 1.44) for the middle tertile of Cd levels and 1.36 (95%: CI: 0.98, 1.90) for the top tertile compared with the bottom tertile - a significant interaction between fetal sex and maternal Cd levels on the risk of GDM (p for interaction=0.03 p for interaction=0.03). Among women carrying male fetuses, the RR of GDM was 1.86 (95% CI: 1.14, 2.93) for the top tertile of Cd levels compared with the bottom tertile - **findings suggest that Cd body burden increases the risk of GDM and that the association may be modified by fetal sex** |
| Oguri et al. 2019 | Japan | Human study  Cross-sectional- study | Association between maternal blood **cadmium and lead** concentrations and gestational diabetes mellitus in the Japan Environment and Children's Study | - n = 16,955 pregnant women - between 2011 and 2014 - Concentrations of Cd and Pb in blood samples collected at 22–28 weeks’ gestation were measured - GDM was diagnosed using the 2011 Japan Society of Obstetrics and Gynecology (JSOG) and Japan Association of Obstetricians and Gynecologists (JAOG) criteria | - Blood Cd and Pb concentrations were slightly higher among women with GDM than among those without GDM; however, these differences were not statistically significant - Elevated blood Cd and Pb concentrations were not associated with increased GDM risk in the nulliparous group (Cd OR 0.76; 95% CI 0.28–2.08 for high vs low category; Pb OR 2.51; 95% CI 0.72–8.72) or the parous group (Cd OR 0.64; 95% CI 0.29–1.44; Pb OR 0.31; 95% CI 0.04–2.29). - **Cd and Pb exposure, in the range of blood levels observed, has no significant relationship with the development of GDM** |
| Romano et al. 2019 | USA | Human study  Prospective Cohort-study | Maternal **urinary cadmium**, glucose intolerance and gestational diabetes in the New Hampshire Birth Cohort Study | - n= 917 - from 2009 – 2017 - Cadmium concentrations were measured in spot urine samples collected at 24–28 weeks gestation - Women were classified as normal (n = 815), glucose intolerant (n = 86), or GDM (n = 16) - Data on GCT ans OGTT were used | - Little to no association was observed for glucose intolerance (OR = 1.11, 95%CI 0.85–1.45) or GDM (OR = 0.86, 95% CI 0.51–1.44) with a doubling of urinary cadmium as compared to normal women - combined outcome of gestational hyperglycemia yielded similar results (OR = 1.07, 95% CI 0.84–1.35). - when stratified by pre-pregnancy body mass index (BMI), there was a slight association with the combined outcome in normal weight women (OR = 1.32, 95% CI 0.88–1.98) and no association in the overweight or obese women - **Cadmium exposure was suggestively associated with increased risk of gestational hyperglycemia among women not already at increased risk of GDM due to being overweight or obese; however, associations of cadmium with gestational hyperglycemia were not statistically significant** |
| Soomro et al. 2019 | France | Human study  nested cross-sectional study | Exposure **to heavy metals** during pregnancy related to gestational diabetes mellitus in diabetes-free mothers | - N= 623 pregnant women - Pb, Cd and Mn were measured in second-trimester blood samples - GDM was diagnosed by using the oral-glucose-tolerance test according to the Carpenter and Coustan criteria | - The prevalences of GDM and IGT were 7.1% and 10.1% respectively - Cd was statistically related to having had a diagnosis of GDM or IGT (Adjusted Odds-Ratio (AOR): 1.61, 1.05–2.48), and Pb to GDM at borderline significance (AOR: 1.65, 0.82–3.34) (p<0,05) - **The results support the role of maternal exposure to heavy toxic metals that persist longtime in the environment as a risk factor for GDM** |
| Xing et al. 2018 | China | Human study  retrospective Cohort study | Relation between **cadmium** exposure and gestational diabetes mellitus | - N= 6837 pregnant women - From 2012-2014 - Information on GDM diagnosis was derived from medical records - Women were diagnosed with GDM by obstetricians based on the IADPSG's criteria - Cd concentrations were measured in urine samples 3 days before delivery using inductively coupled plasma mass spectrometry | - For about 3-fold increase in Cd concentrations, there were 16% [relative risk (RR) =1.16; 95% confidence interval (CI): 1.03, 1.33] increase in risk of GDM - Compared with women in the lowest quartile of urinary Cd levels, women in the highest quartile had 1.30 higher risk of GDM [95% CI: 1.05, 1.61; p-trend <0.05]. - overweight/obese women with higher urinary Cd levels had significantly higher risk of GDM, compared with women in the reference category of lowest quartile of Cd and normal pre-pregnancy body mass index [RR =2.71; 95% CI: 1.81, 4.07]. - **Results presented a significantly positive association between urinary Cd levels and risk of GDM, supporting the hypothesis that environmental exposure to Cd may contribute to the development of GDM** |

**Table 7: Overview of included studies: Season**

| Author, Year | Location | Study design | Topic | Subjects (n, characteristics) | Main findings |
| --- | --- | --- | --- | --- | --- |
| Katsarou et al. 2016 | Sweden | Human study  Retrospective Cohort study | **Seasonal** Pattern in the Diagnosis of Gestational Diabetes Mellitus in Southern Sweden | - n= 11 538 women in the twenty-eighth week of pregnancy - during the years 2003–2005 - GDM was diagnosed by using OGTT and a slight modification of the diagnostic criteria by the WHO | - seasonal frequency of GDM ranged from 3.3% in spring to 5.5% in summer (p<0,0001) - Mean 2-h glucose concentrations followed the same seasonal trend, with a difference of 0.15 mmol/L between winter and summer (p<0,0001) - summer (June–August) was associated with increased 2-h glucose level ( p<0,0001) and increased frequency of GDM compared to the other seasons (odds ratio 1.51, 95% confidence interval 1.24–1.83, and ). - **findings suggest seasonal variation in the 2-h glucose concentration in the OGTT and in the proportion of women diagnosed with GDM, with a peak in the summer** |
| Meek et al. 2020 | UK | Human study  retrospective Cohort study | **Seasonal** variations in incidence and maternal–fetal outcomes of gestational diabetes | - n= 985 pregnant women - women were screened for GDM with a random plasma glucose check, GCT and OGTT - temperature, dew point, humidity atmospheric pressure, mean wind speed, sunshine, rainfall, and maximum wind speed were recorded by the Cambridge Digital Technology Group weather station | - The incidence of gestational diabetes varied by 30% from peak incidence (October births) to lowest incidence (March births; P=0.031). - Ambient temperature at time of testing (28 weeks) was strongly positively associated with diagnosis (P<0.001). - Significant seasonal variation was evident in birth weight in gestational diabetes‐affected pregnancies (average 54th centile June to September; average 60th centile December to March; P=0.027) - **There is substantial seasonal variation in gestational diabetes incidence and maternal–fetal outcomes, even in a relatively cool temperate climate** |
| Moses et al. 2016 | Australia | Human study  Cross-sectional-study | **Seasonal** changes in the prevalence of gestational diabetes mellitus | - N= 7369 pregnant women - all women not known to have HIP are tested at 24–28 weeks gestation with a GTT | - In winter, the median 1-h and 2-h glucose results after GTT were significantly (P < 0.0001) lower than the overall 1-h and 2-h results - The prevalence of GDM at the 1-h diagnostic level was 29% higher in summer and 27% lower in winter than the overall prevalence (P = 0.02). - The prevalence of GDM at the 2-h diagnostic level was 28% higher in summer and 31% lower in winter than the overall prevalence (P = 0.01) - **The prevalence of GDM varies according to seasons, which leads to the possible overdiagnosis of GDM in summer and/or underdiagnosis in winter** |
| Petry et al. 2019 | UK | Human study  Prospective Cohort study | **Temporal trends** without seasonal effects on gestational diabetes incidence relate to reductions in indices of insulin secretion: the Cambridge Baby Growth Study | - N= 1074 pregnant women - who underwent oral glucose tolerance tests around week 28 of pregnancy - Between 2001-2009 - OGTT glucose concentrations (i.e. ≥ 5.1 and 10.0 mmol/L, respectively) were used to define the presence of GDM. - Insulin sensitivity and pancreatic β-cell function were estimated using the homeostasis model assessment (HOMA S and B, respectively) - temporal and seasonal trends were considered | - There was a temporal increase in gestational diabetes incidence over the course of recruitment to this study [0.014 (0.005, 0.022) proportional increase per year, p = 2.1 × 10−3], but no seasonal effect (p = 0.7) - There was, no significant association of GDM with seasonality [amplitude: 9.8 (− 14.7, 34.3), p = 0.4; acrophase − 0.6 (− 3.0, 1.9), p = 0.7] (Fig. 1). Cosinor analysis revealed no significant association of the OGTT fasting glucose concentration with seasonality [amplitude: 21.3 (− 24.1, 66.6), p = 0.4; acrophase − 0.4 (− 2.4, 1.6), p = 0.7]. - The insulin disposition index was not associated with season of testing [amplitude: 26.0 (− 38.5, 90.5), p = 0.4; acrophase 1.1 (− 1.5, 3.7), p = 0.4]. The c-peptide disposition index was also negatively associated with year of testing (β′ = − 0.074, p = 0.03, n = 882). - **there were** **temporal, but not seasonal, increases in gestational diabetes incidence between the years 2001 and 2009, which appeared to be related more to reductions in insulin secretion than sensitivity**. |
| Shen et al. 2019 | Australia | Human study  Cohort study | **Seasonality, temperature** and pregnancy oral glucose tolerance test results in Australia | - n= 2120 pregnant women - with available OGTT results between 24 to 32 weeks gestation - between 2001 – 2006 - Fasting plasma glucose, 1-h plasma glucose, 2-h plasma glucose, HbA1c, HOMA-IR, and umbilical cord C-peptide and glucose values were categorized by season and correlated to monthly temperature records | - Small but significant (p < 0.01 on ANOVA) elevations in fasting glucose (+ 0.12 mM), HbA1c (+ 0.09%), and HOMA-IR (+ 0.88 units) were observed during the winter months - Conversely, higher 1-h (+ 0.19 mM) and 2-h (+ 0.33 mM) post-load glucose values (both p < 0.01) were observed during the summer months - Overall prevalence of GDM did not display significant seasonal variations due to the opposing trends seen in the fasting versus 1-h and 2-h post-load values (p=0,009) - **A significant winter increase was observed for fasting plasma glucose, HbA1c, and HOMA-IR, which contrasted with changes in 1-h and 2-h post-load venous plasma glucose values, while overall prevalence of GDM did not vary significantly by seasons** |
| Verburg et al. 2016 | Australia | Human study  retrospective cohort study | **Seasonality** of gestational diabetes mellitus: A South Australian population study | - N= 60306 pregnant women - Between 2007-2011 - Data on GDM based on universal screening for GDM (GCT and OGTT) - Seasonality of GDM was initially investigated using a univariate logistic regression analysis using eDoC divided in months as predictor | - During the study period, 3632 (6.0%) women were diagnosed with GDM - Seasonal modeling showed a strong relation between GDM and eDoC in july and august (p<0.001) - Unadjusted and adjusted models (adjusted for maternal age, body mass index (BMI), parity, ethnicity, socioeconomic status, and chronic hypertension) demonstrated the presence of a peak incidence occurring among pregnancies with estimated date of conception (eDoC) in winter (June/July/August), with a trough for eDoc in summer (December/January/February) - **Several maternal lifestyle and psychosocial factors associated with seasonality and GDM may be influential in the pathophysiologic mechanisms of GDM** |
| Wainstock et al. 2019 | Israel | Human study  Retrospective Cohort study | Pregnant women may be sweeter in the summer: **Seasonal changes** in glucose challenge tests results. A population-based study | - N= 101.359 fasting glucose levels and GCT results - Between 2005 and 2016 - glucose challenge test (GCT) and OGTT results were analysed - Dates of tests results were recorded according to calendric months, categorized into 4 seasons | - Mean GCT was 110 ± 28.9 mg/dL, and 14.5% (n = 14,652) were pathological - Both the mean and the incidence of pathological GCT were lowest in the winter, followed by spring, fall, and summer, (p for trend < 0.001). - The difference in mean GCT between winter and summer was 7.82 ± 0.24 mg/dL (95% CI, 7.35–8.29). - After adjustment for BMI and age, **having a GCT in the winter was independently associated with the lowest risk for pathological GCT, as compared to all other seasons** |
| Wang et al. 2020 | Taiwan | Human study  Retrospective Cohort study | **Seasonality** of gestational diabetes mellitus and maternal blood glucose levels: Evidence from Taiwan | - N= 6396 pregnant women - Between 2012-2014 - included women underwent the 2-step approach for GDM screening and were diagnosed with GDM based on the Carpenter-Coustan criteria - effect of season on GDM diagnosis was estimated | - During the study period, 418 (6.5%) pregnancies were diagnosed as GDM - The model demonstrated an increased prevalence of GDM in spring and summer (odds ratio: 1.59, 95% confidence interval: 1.13–2.24; odds ratio: 1.59, 95% confidence interval: 1.14–2.23, respectively) compared to winter (p=0,02) - the model demonstrated an increase of 2.56 mg/dL glucose in the 50-g glucose challenge test in summer compared to winter. In glucose challenge test-positive pregnancies, the season also had an effect on the results of the 100-g 1-h, 2-h, and 3-h oral glucose tolerance tests, but no effect on the 100-g fasting oral glucose tolerance tests (p<0,05) - **GDM prevalence in Taiwan presents** **seasonal variation, with the highest risk during spring and summer due to post-glucose load level variations**. |

**Table 8: Overview of included studies: Ambient temperature**

| Author, Year | Location | Study design | Topic | Subjects (n, characteristics) | Main findings |
| --- | --- | --- | --- | --- | --- |
| Molina-Vega et al 2020 | Spain | Human study  Retrospective cohort study | Relationship between environmental temperature and the diagnosis and treatment of gestational diabetes mellitus: An observational retrospective study | - N= 2374 women - From 2015 – 2016 - GDM diagnosis was performed using a two-step strategy according to National Diabetes Data Group (NDDG) criteria - The mean temperature and the temperature change (the difference between the maximum and minimum temperature) for the day of the OGTT and for each of the preceding 14 and 28 days was determined from the Local Meteorological Agency | - GDM was diagnosed in 473 patients - We found significant seasonal differences in the percentage of GDM: 24.4% in summer vs. 15.6% in autumn (p < 0.01). - odds ratio (OR) for being diagnosed with GDM was 1.78 in summer relative to autumn, after controlling for age - A higher mean temperature the day of the OGTT and the preceding 14 and 28 days increased the risk of being diagnosed with GDM the months in which temperature was rising (March–August) but not the months in which temperature was decreasing (September–February) - Neither the season nor the environmental temperature affected the risk of requiring insulin therapy - **There is a higher prevalence of GDM diagnosis at warmer seasons and at rising temperatures the 2–4 weeks prior to the OGTT** |
| Retnakaran et al. 2018 | Canada | Human study  prospective observational cohort study | Impact of daily incremental change in **environmental temperature** on beta cell function and the risk of gestational diabetes in pregnant women | - N= 318 pregnant women - Blood glucose, beta cell function and insulin sensitivity were evaluated in relation to 9 temperature change variables on certain days before the OGTT (7, 14, 21, 28, 35, 42, 49 and 56 days) - All women were screened for GDM by GCT and OGTT - Insulin sensitivity was measured by Matsuda index and HOMA-IR - Beta cell function was assessed by ISSI-2 and (IGI)/HOMAR-IR | - Temperature changes in the preceding 14, 21, 28, 35, 42, 49 and 56 days (rather than mean temperatures) emerged as independent predictors of blood glucose - These relationships were evident in months where mean daily temperature was rising (February - July), but not in those where it was falling (August - January) - in February - July, the temperature changes in the preceding 21, 28 and 35 days emerged as predictors of both poorer beta cell function and higher blood glucose - in February - July, the changes in temperature in the preceding 21 days (OR 1.16, 95% CI 1.01, 1.33) and 28 days (OR 1.20, 95% CI 1.03, 1.39) were independent predictors of GDM, while mean temperatures were not - **In pregnant women, rising environmental temperature in the 3-4 weeks prior to glucose tolerance testing may be associated with beta cell dysfunction and an increased risk of GDM** |
| Su et al. 2020 | Taiwan | Human study  population-based cohort study | A population-based study on the prevalence of gestational diabetes mellitus in association with **temperature** in Taiwan | - N= 43.538 women diagnosed with GDM - Between 2013 – 1014 - GDM was diagnosed according both Carpenter and Coustan's criteria and the criteria proposed by the International Association of the Diabetes and Pregnancy Study Group - mean daily temperature and difference in temperature within a day was calculated over a 35-day period prior to GDM diagnosis or the first day of the 27th gestational week rovided by the Central Weather Bureau of Taiwan | - summer and fall were associated with higher risk of GDM diagnosis, with aOR [95% CI] of 1.05 [1.04-1.07] and 1.04 [1.02-1.06] in reference to winter - an increase of 1 °C from 14 °C to 27 °C was associated with an aOR of 1.03 [1.02-1.03]. - The aOR greatly increased to 1.54 [1.48-1.60] after 28 °C. An increase of 1 °C difference within a day was associated with a reduced aOR at 0.90 [0.87-0.92]. - **A higher prevalence of GDM was associated with a higher daily temperature, but with a smaller difference in temperature within a day.** |
| Vasileiou et al. 2018 | Greece | Human study  Retrospective and prospective study | The impact of **environmental temperature** on the diagnosis of gestational diabetes mellitus | Retrospective study:   - N= 7618 pregnant women who underwent a 3-h 100 g OGTT during the 3rd trimester of gestation - Between 2000 and 2012 - mean month temperature during the duration of the study were obtained from the Hellenic National Meteorological Service   Prospective study:   - N= 768 pregnant women tested in the 3rd trimester of gestation with a 75 g OGTT. - Temperature was recorded every day at 09:00 h | Retrospective study:   - GDM prevalence differed significantly by season: winter = 28.1%, summer = 39.2%, spring = 32.4% and autumn = 32.4% (P < 0.0001) - The odds ratio for being diagnosed with GDM was much higher during summer 1.65 (95% CI: 1.43–1.90), with spring and autumn following with 1.23 (95% CI: 1.08–1.39) compared to winter - Glucose levels during OGTT were measured: significantly increased blood glucose values were observed at 60, 120 and 180 min in summer, which remained significant after adjustment for age, gestational age, BMI, weight gain during pregnancy and blood pressure   Prospective study:   - At temperatures above 25°C, the average glucose 60-min and 120-min levels were increased - The relative risk for abnormal glucose values at 60 min, when the environmental temperature increased over 25°C, was 2.2 (1.5–3.3). - **GDM prevalence in Greece presents seasonal variation, with higher risk during summer due to post glucose load level variations** |
| Zhang et al. 2021 | China | Human study  Prospective Cohort study | Assessing the effects of non-optimal **temperature** on risk of gestational diabetes mellitus in a cohort of pregnant women in Guangzhou, China | - N= 5165 pregnant women (13 to 50 years old) - Between 2011-2014 - Daily maximum (Tmax), minimum temperature (Tmin) and diurnal temperature range (DTR) were obtained - Criteria of the National Health Commission of the People’s Republic of China was used to diagnose GDM | - 604 women were diagnosed with GDM (11.7%) - Compared with a reference temperature (50th percentile of Tmax), we found that extreme high temperature (99th percentile of Tmax) exposure during 21st and 22nd gestational weeks was associated with an increased risk of GDM - Extreme low temperature (1st percentile of Tmax) exposure during 14th to 17th weeks increased the risk of GDM (p<0,05) - per 1 °C increment of DTR during 21st to 24th weeks was associated with an elevated GDM risk - **non-optimal temperature is an independent risk factor of GDM. The time window of susceptibility for extreme temperatures and DTR exposure on the risk of GDM generally occurred in second trimester of pregnancy** |

**Table 9: Overview of included studies: Air pollution**

| Author, Year | Location | Study design | Topic | Subjects (n, characteristics) | Main findings |
| --- | --- | --- | --- | --- | --- |
| Choe et al. 2019 | USA | Human study  Cross-sectional-study | **Ambient air pollution** during pregnancy and risk of gestational diabetes in New York City | - N = 256,372 pregnant women - Between 2008-2010 - GDM was assessed on the presence of clinical records - integrated air pollution samples were collected at 150 monitoring sites in each of the four seasons for one 2-week session and in every 2-week period at five reference locations to track city-wide temporal variation | - 17,065 women were identified as having GDM - PM2.5 in the 2nd trimester (OR: 1.06, 95% CI: 1.02, 1.10 per interquartile range increase in PM2.5), and NO2 in the 1st trimester (OR: 1.05, 95% CI: 1.01, 1.09) **were associated with higher odds of GDM. These positive associations were robust to different model specifications** - The association between 2nd trimester PM2.5 and GDM was more pronounced in women who were overweight or obese, younger, and not on Medicaid |
| Choe et al. 2018 | USA | Human study  Retrospective cohort-study | **Air pollution**, land use, and complications of pregnancy | - N= 61,640 mother infant pairs (>18 years) - residential levels of ambient fine particulate matter (PM2.5) and black carbon (BC) was estimated using spatial-temporal geostatistical models - GDM was identified from birth certificate data and ICD-9 codes obtained from clinical records. GDM was considered as present if ICD-9648.8× was listed, and absent otherwise - Other outcomes were gestational hypertension, and preeclampsia | - 2nd trimester PM2.5 (OR = 1.08, 95% CI: 1.00, 1.15 per interquartile range increase in PM2.5) and living close to a major roadway (1.09, 95% CI: 1.00, 1.19) were associated with higher odds of GDM - **risk of GDM was positively associated with PM2.5 and proximity to busy roadways**, and negatively associated with proximity to blue space, highlighting the importance of the natural and built environment to maternal health |
| Dastoorpoor et al. 2021 | Iran | Human study  Ecologic and population based Cohort study | Prenatal exposure **to ambient air pollution** and adverse pregnancy outcomes in Ahvaz, Iran: a generalized additive model | - n= 37,000 births - between 2008 - 2018 - Data on adverse pregnancy outcomes and air pollutants including ozone (O3), nitric oxide (NO), nitrogen dioxide (NO2), sulfur dioxide (SO2), carbon monoxide (CO), particles with a diameter of less than 10 µm (PM10) and particles with a diameter less than 2.5 µm (PM2.5) were inquired - from the Health Department of Ahvaz Jundishapur University of Medical Sciences and the Environmental Protection Agency of Khuzestan Province - spontaneous abortion, preeclampsia, macrosomia, stillbirth and gestational hypertension were other outcomes | - During study period 3953 cases of gestational diabetes were reported - **NO and NO2 pollutants showed significant and direct associations with gestational diabetes (p<0,01)** - SO2 pollutant on 0, 1, 3, 4, and 6-day lags and PM10 on lag 0 had direct and significant associations with spontaneous abortion - The highest relative risk levels for NO was on lag 0 with RR = 1.012 (95% CI 1.002–1.021), ­NO2 on lag 0 with RR = 1.009 (95% CI 1.002–1.017) and ­PM25 on lag 3 with RR = 1.002 (95% CI 1.001–1.004). - ­SO2 and ­O3 showed inverse and significant associations with gestational diabetes. But, CO and ­PM10 did not show any significant association |
| Fleisch et al. 2014 | USA | Human study  Prospective Cohort study | **Air pollution** exposure and abnormal glucose tolerance during pregnancy: the project Viva cohort | - N= 2,093 pregnant women - From 1999 - 2002 - impaired glucose tolerance (IGT) and gestational diabetes mellitus (GDM) was identified according the American Diabetes Association (ADA) criteria - second-trimester PM2.5 and black carbon exposure at each woman’s residential address using spatiotemporal models were estimated - neighborhood traffic density was estimated using the 2002 road inventory from the Massachusetts Executive Office of Transportation | - prevalence of IGT was elevated in the highest (vs. lowest) quartile of exposure to spatiotemporal PM2.5 [odds ratio (OR) = 2.63; 95% CI: 1.15, 6.01] and traffic density (OR = 2.66; 95% CI: 1.24, 5.71). - IGT also was positively associated with other exposure measures, although associations were not statistically significant. - No pollutant exposures were positively associated with GDM. - **Greater exposure to PM2.5 and other traffic-related pollutants during pregnancy was associated with IGT but not GDM** |
| Fleisch et al. 2016 | USA | Human study  Prospective Cohort study | **Air pollution** exposure and gestational diabetes mellitus among pregnant women in Massachusetts: a cohort study | - N= 159,373 pregnant women - from 2003-2008 without pre-existing diabetes - women were diagnosed woth GDM according the American Diabetes Association (ADA) criteria - maternal GDM designation from the birth records were obtained - first and second trimester residential particulate (PM2.5) exposure at woman’s residential address at the time of delivery were estimated - geographic information systems were used to estimate neighbourhood traffic density | - Of 159,373 women, 5,381 (3.4 %) developed GDM - None of the exposures were associated with GDM in the full cohort [e.g. OR 0.99 (95 % CI: 0.95, 1.03) for each interquartile range (IQR) increment in second trimester PM2.5]. - women less than 20 years had 1.36 higher odds of GDM (95 % CI: 1.08, 1.70) for each IQR increment in second trimester PM2.5 exposure - **greater exposure to PM2.5 during the second trimester was associated with GDM** |
| Hehua et al. 2021 | China | Human study  retrospective Cohort study | Dietary patterns and associations between **air pollution** and gestational diabetes mellitus | - N= 2244 pregnant women - From 2018 – 2019 - GDM was diagnosed based on the results of the 75-g oral glucose tolerance test completed during 24–28 weeks of gestation according to the 2010 recommendations of the International Association of Diabetes and Pregnancy Study Groups - Pre-pregnancy dietary intake was assessed using a validated interviewer-administered Food Frequency Questionnaire - Daily air pollution data for PM2.5, PM10, SO2, NO2, CO, and ozone (O3) from 2017 to 2019 were obtained from 78 environmental monitoring stations located in various places in Liaoning province | - long-term exposure to nitrogen dioxide (NO2) and carbon monoxide (CO) before pregnancy was significantly associated with an increased risk of GDM, the animal foods pattern significantly modified these associations (p<0,05) - compared with a lower intake in the animal foods pattern (NO2, odds ratio [OR] = 1.07, 95% confidence interval [CI]: 0.84, 1.35; CO, OR = 1.05, 95% CI: 0.81, 1.34), higher intake in the animal foods pattern (NO2, OR = 1.41, 95% CI: 1.09, 1.83; CO, OR = 1.36, 95% CI: 1.05, 1.76) before pregnancy increased the hazardous effects of NO2 and CO on GDM development. - **there was a significant interaction effect between animal foods pattern and exposure** |
| Hu et al. 2015 | USA | Human study  Prospective Cohort study | Association of **Atmospheric Particulate Matter and Ozone** with Gestational Diabetes Mellitus | - n= 410,267 pregnant women - between 2004 and 2005 - GDM was diagnosed based on the results of the 75-g oral glucose tolerance test completed during 24–28 weeks of gestation according to the 2010 recommendations of the International Association of Diabetes and Pregnancy Study Groups - Air pollution exposure data was obtained from the U.S. EPA and CDC’s National Environmental Public Health Tracking Network - EPA provided the HBM data from 2001 to 2008 for two air pollutants, PM2.5 and O3 | - increased odds of GDM with per 5-μg/m3 increase in PM2.5 (ORTrimester1 = 1.16; 95% CI: 1.11, 1.21; ORTrimester2 = 1.15; 95% CI: 1.10, 1.20; ORPregnancy = 1.20; 95% CI: 1.13, 1.26) and per 5-ppb increase in O3 (ORTrimester1 = 1.09; 95% CI: 1.07, 1.11; ORTrimester2 = 1.12; 95% CI: 1.10, 1.14; ORPregnancy = 1.18; 95% CI: 1.15, 1.21) during both the first trimester and second trimester as well as the full pregnancy in single-pollutant models. - the ORs for PM2.5 during the first trimester and the full pregnancy were attenuated, and no association was observed for PM2.5 during the second trimester in the co-pollutant model (OR = 1.02; 95% CI: 0.98, 1.07 - **exposure to air pollution during pregnancy is associated with increased risk of GDM** |
| Hu et al. 2021 | China | Human study  retrospective Cohort study | Association of **ambient particle pollution** with gestational diabetes mellitus and fasting blood glucose levels in pregnant women from two Chinese birth cohorts | - n= 2326 pregnant women - between 2016 and 2018 - PM10, PM2.5 and black carbon (BC) exposure concentrations in the first and second trimesters of pregnancy were collected - GDM was diagnosed according to the Chinese Obstetricians and the Gynecologists Association diagnostic criteria | - positive and significant associations of PM10, PM2.5 and BC exposure with fasting glucose, particularly in the second trimester was observed (p<0,05) - PM10, PM2.5 and BC were strongly correlated and displayed similar cumulative (lag 0–3 weeks) associations with fasting blood glucose - **exposure to air pollution during pregnancy exerts cumulative, adverse effects on fasting blood glucose** |
| Jo et al. 2019 | USA | Human study  Retrospective cohort study | Associations of gestational diabetes mellitus with **residential air pollution** exposure in a large Southern California pregnancy cohort | - n= 239,574 pregnancies - between 1999 and 2009 - the American College of Obstetricians and Gynecologists guidelines for GDM screening were used - Concentrations of ambient nitrogen dioxide (NO2), particulate matter (PM) ≤2.5 μm in aerodynamic diameter (PM2.5) and ≤10 μm (PM10), and ozone (O3) during preconception and the first trimester of pregnancy at the residential birth address were estimated from data compiled from the EPA regional air quality monitoring network | - In single-pollutant models, preconception NO2 was associated with increased risk of GDM (OR = 1.10 per 10.4 ppb, 95% confidence interval [CI]: 1.07, 1.13). - First trimester NO2 was weakly associated with GDM, and this was not statistically significant (OR = 1.02 per 10.4 ppb, 95% CI: 0.99, 1.05). - In single-pollutant models, preconception PM2.5 and PM10 associations were associated with increased risk of GDM (OR = 1.04 per 6.5 μg/m3, 95% CI: 1.01, 1.06; OR = 1.03 per 16.1 μg/m3, 95% CI: 1.00, 1.06, respectively), but these effect estimates were not robust to adjustment for other pollutants - In single-pollutant models, preconception and first trimester O3 were associated with reduced risk of GDM (OR = 0.94 per 15.7 ppb, 95% CI: 0.92, 0.95; OR = 0.95 per 15.7 ppb, 95% CI: 0.94, 0.97), associations that were robust to adjustment for co-pollutants - **Maternal exposure to NO2 during the preconception trimester may increase risk of GDM.** |
| Kang et al. 2020 | China | Human study  Prospective cohort study | Associations of exposure to **fine particulate matter** during pregnancy with maternal blood glucose levels and gestational diabetes mellitus: Potential effect modification by ABO blood group | - N = 4783 pregnant women - Daily PM2.5 exposure levels for each woman during pregnancy were estimated by national environmental surveillance stations - All women completed the routine clinical screening for GDM through a OGTT - GDM was diagnosed based on the recommendation of the International Association of Diabetes and Pregnancy Study Groups | - Among all the 4783 participants, 394 (8.24%) had GDM - Exposure to PM2.5 was found to be positively associated with elevated fasting glucose level during the whole study period [0.382 mg/dL, 95% confidence interval (CI): 0.179-0.586, per 10 μg/m3 increase in PM2.5], the first trimester (0.154 mg/dL ,95% CI: 0.017-0.291) and the second trimester (0.541 mg/dL, 95% CI: 0.390-0.692) (p<0,05) - Increased risks of GDM for each 10 μg/m3 increase in PM2.5 levels were observed during the whole study period [relative risk (RR): 1.120, 95% CI: 1.021-1.228] and the first trimester (RR: 1.074, 95% CI: 1.012-1.141), but not the second trimester (RR: 1.035, 95% CI: 0.969-1.106) - **results enriched epidemiological evidence linking PM2.5 exposures during pregnancy to elevated maternal glucose levels and increased risk of GDM. More importantly, we first highlighted that the impact of PM2.5 on GDM might be greater among pregnant women with blood group A** |
| Li et al. 2020 | China | Human study  Cross-sectional-study | **Air pollutants** concentration and variation of blood glucose level among pregnant women in China: A cross-sectional study | - n= 32,963 pregnant women - between 2013-2017 - associations of air pollution concentration with blood glucose was examined - GCT and OGTT were performed to consider blood glucose levels and to diagnose GDM - Seven fixed monitoring stations measured data on hourly air pollution and weather. | - increase in PM2.5 concentration during the second trimester was associated with 2.09% (7-day lag, 95% CI: 1.62%, 2.57%) higher GCT blood glucose levels; the corresponding figures for PM10, NO2 and CO were 3.99% (7-day lag, 95% CI: 3.28%, 4.71%), 7.40% (6-day lag, 95% CI: 6.41%, 8.39%), and 4.23% (4-day lag, 95% CI: 3.75%, 4.71%), respectively - For PM2.5, PM10 and NO2 concentrations, significant associations were observed among women with older age, overweight/obesity, and lower education level (p<0,05) - pregnant women with lower BMI were more susceptible to higher CO-related GCT blood glucose levels - **women had higher blood glucose levels when exposed to higher residential PM2.5, PM10, NO2 and CO during their pregnancy. Women with higher BMI and lower education levels were more susceptible to the adverse effects of ambient air pollution** |
| Lin et al. 2020 | China  Birth cohort study | Human study  Cross-sectional-study | **Ambient air pollution** exposure associated with glucose homeostasis during pregnancy and gestational diabetes mellitus | - N= 12,842 pregnant women - Air pollutant (fine particulate matter (PM2.5), particulate matter with an aerodynamic diameter of 10 μm or less (PM10), sulfate dioxide (SO2), nitrogen dioxide (NO2) and ozone (O3)) concentrations from the air monitoring stations in Foshan were used to estimate individual air pollutant exposure during the first two trimesters - Women were screened for GDM through an OGTT between late trimester 2 and trimester 3. | - 3055 (23.8%) women had GDM - A 10 μg/m3 increase in PM2.5, PM10 and SO2 during trimester 1, trimester 2 and two trimesters were associated with 0.07 mmol/L to 0.29 mmol/L increment in OGTT-fasting glucose levels in single-pollutant model - A 10 μg/m3 increase in NO2 and O3 during two trimesters were associated with 0.15 mmol/L and 0.12 mmol/L decrease in OGTT-fasting glucose in single-pollutant model. - no significant or weaker effects of O3 during two trimesters on OGTT-fasting glucose were observed in two-pollutant models - **exposure to PM2.5, PM10 and SO2 were associated with increased risk of GDM in both single- and two-pollutant models.** |
| Lu et al. 2017 | Taiwan | Human study  Retrospective cohort study | Association of temporal distribution of **fine particulate matter** with glucose homeostasis during pregnancy in women of Chiayi City, Taiwan | - n= 3589 non-diabetic pregnant women - Between 2006-2014 - GCT and OGTT were performed based on the recommendation of the International Association of Diabetes and Pregnancy Study Groups - PM2.5 and other air pollution data were obtained from one fixed-site monitoring station - daily average air pollution and weather data were measured and matched these data with the women's OGTT measurement dates | - There were significant relationships between PM2.5 and the glucose homeostasis indicators, including fasting, 1-h, 2-h, and 3-h glucose levels in the single-pollutant covariate-adjusted model (p<0,05) - pre-screening 1-month to 1-year moving averages of IQR increases in PM2.5 were significantly associated with elevated fasting OGTT glucose levels (1.32–5.87 mg/dL). - **PM2.5 exposure in the second trimester may enhance positive associations between PM2.5 and OGTT glucose levels during pregnancy. Exposure to PM2.5 was associated with glucose homeostasis during pregnancy.** |
| Malmqvist et al. 2013 | Sweden | Human study  Retrospective cohort study | Gestational diabetes and preeclampsia in association with **air pollution** at levels below current air quality guidelines | - N= 81,110 pregnant women - Between 1999-2005 - Women underwent GDM screening and is diagnosed based on plasma glucose > 10 mmol/L 2 hr after oral administration of 75 g of glucose - Modelled exposure to nitrogen oxides (NOx), expressed as mean concentrations per trimester, and proximity to roads of different traffic densities were used as proxy indicators of exposure to combustion-related air pollution | - The prevalence of gestational diabetes increased with each NOx quartile, with an adjusted odds ratio (OR) of 1.69 (95% CI: 1.41, 2.03) for the highest (> 22.7 µg/m3) compared with the lowest quartile (2.5–8.9 µg/m3) of exposure during the second trimester - Both outcomes were associated with high traffic density, but ORs were significant for gestational diabetes only. - **NOx exposure during pregnancy was associated with gestational diabetes and preeclampsia in an area with air pollution levels below current air quality guidelines** |
| Melody et al. 2020 | Australia | Human study  Prospective cohort study | Maternal exposure to **fine particulate matter** from a large coal mine fire is associated with gestational diabetes mellitus: A prospective cohort study | - N= 3612 pregnant women with singleton pregnancies - Between 2012 – 2015 - Women were screened for GDM between 24- and 28- weeks gestation with an OGTT - Diagnostic threshold was lowered during the study period to align with the WHO- 2013 diagnostic criteria, - Average and peak fine particulate matter (PM2.5) was assigned to residential address at delivery using a chemical transport model - minimum and maximum daily temperature data were obtained from the Australian Government Bureau of Meteorology for the Morwell station | - 766 women were exposed to the smoke event - Average maternal PM2.5 exposure was 4.4 μg/m3 (SD 7.7; IQR 2.12). Average peak PM2.5 exposure was 44.9 μg/m3 (SD 57.1; IQR 35.0) - An interquartile range increase in peak PM2.5 was associated with a 16% increased likelihood of gestational diabetes mellitus (95%CI 1.09, 1.22; <0.0001) - **an interquartile range increase in average PM2.5 was associated with a 7% increased likelihood of gestational diabetes mellitus (95%CI 1.03, 1.10; <0.0001).** |
| Najafi et al. 2020 | Iran | Human study  Cross-sectional-study | Preconception **air pollution** exposure and glucose tolerance in healthy pregnant women in a middle-income country | - n= 250 pregnant women - in summer 2019 - glucose tolerance was determined according to the American Diabetes Association (ADA) (2008) criteria - Land use regression (LUR) models were applied to estimate the annual mean of PM1, PM2.5 and PM10 at the residential address - Traffic indicators, including proximity of women to major roads as well as total streets length in 100, 300 and 500 m buffers around the home were calculated | - Exposure to PM1, PM2.5 and PM10 was significantly associated with higher FBG concentration - Higher total streets length in a 100 m buffer was associated with higher FBG and 1-h glucose concentrations - An interquartile range (IQR) increase in proximity to major roads was associated with a decrease of − 3.29 mg/dL (95% confidence interval (CI): − 4.35, − 2.23, P-value < 0.01) in FBG level and − 3.65 mg/dL (95% CI, − 7.01, − 0.28, P-value = 0.03) decrease in 1-h post-load glucose - **higher preconception exposure to air pollution was associated with higher FBG and 1-h glucose concentrations during pregnancy** |
| Padula et al. 2019 | USA | Human study  Cross-sectional-study | Prenatal exposure to **air pollution**, maternal diabetes and preterm birth | - n= 262,182 pregnant women - between 2000 – 2006 - Ambient air quality data were acquired from U.S. Environmental Protection Agency’s Air - Quality System database - carbon monoxide (CO), nitrogen dioxide (NO2), particulate matter ≤ than 10 μm (PM10), and PM ≤ than 2.5 μm (PM2.5) were calculated - Air pollutants were dichotomized at the highest quartile and compared to the lower three quartiles | - There were consistent inverse associations between exposure to air pollution during the first two trimesters and gestational diabetes (statistically significant odds ratios (OR) less than 1). - When stratified by any diabetes (gestational or pre-existing), associations between air pollution exposure during pregnancy and categories of preterm birth (20–27, 28–31, 32–33, 34–36 weeks) were generally similar with few exceptions of exposures to carbon monoxide (CO) and particulate matter < 2.5 µm (PM2.5). - **The associations between traffic-related air pollution and gestational diabetes were in the unexpected (“protective”) direction**. Among those with any diabetes, associations were stronger between CO and PM2.5 and extremely preterm birth |
| Pan et al. 2017 | Taiwan | Human study  Cohort study | Gestational diabetes mellitus was related to **ambient air pollutant** nitric oxide during early gestation | - N= 19.606 pregnant women - In 2005 - A structured questionnaire was used to determine the prevalence of GDM - pregnant women were classified as having GDM according to the American Diabetes Association criteria - Air pollution data were collected from 77 fixed-site air monitoring stations in Taiwan during 2004–2006 - At each station, automatic hourly monitoring of the following pollutants occurred: PM10, carbon monoxide, NO, NO2, and NOx; SO2 and O3 | - 378 (1.9%) had been diagnosed as having GDM. These women were older and had higher BMIs than the women without GDM - The risks of GDM onset were significantly associated with NO exposure during the first [adjusted OR (aOR): 1.05, 95% confidence interval (CI): 1.02–1.08] and second (aOR: 1.05, 95%CI: 1.02–1.08) trimesters. - Under the two-pollutant model, the effect of NO exposure was also significant during the first (aOR: 1.05, 95%CI: 1.02–1.08) and second (aOR: 1.05, 95%CI: 1.02–1.09) trimesters - **The results indicated that exposure to higher NO levels during pregnancy increases the risk of GDM (p<0,05)**. |
| Pedersen et al. 2017 | Denmark | Human study  Cohort study | Gestational diabetes mellitus and exposure to **ambient air pollution** and road traffic noise: A cohort study | - N= 72,745 pregnant women - Between 1997-2002 - diagnostic criteria: the Danish clinical guidelines and the WHO standard - maternal residential address history from conception until delivery, including dates of moving, was collected from the Civil Registration System - nitrogen dioxide (NO2) was evaluated using the advanced AirGIS dispersion model - noise from road traffic was calculated at the most exposed facade of each residential address using SoundPLAN | - a total of 565 and 210 women, respectively, had GDM - A 10-μg/m3 increase in NO2 exposure during first trimester was, however, associated with an increased risk of WHO-GDM (adjusted odds ratio (OR) = 1.24; 95% confidence interval (CI): 1.03, 1.49). - The corresponding OR associated with a 10-dB higher road traffic noise level was 1.15 (0.94 to 1.18). - In mutually adjusted models the OR for NO2 remained similar 1.22 (0.98, 1.53) whereas that for road traffic noise decreased to 1.03 (0.80, 1.32). Significant associations were also observed for exposure averaged over the 2nd and 3rd trimesters and the full pregnancy (p<0,05) - **No risk was evident for the common Danish criterion of GDM. NO2 was associated with higher risk for GDM according to the WHO criterion, which might be due to selection bias** |
| Rammah et al. 2020 | USA | Human study  Retrospective cohort study | **Particle air pollution** and gestational diabetes mellitus in Houston, Texas | - n= 354,328 pregnant women - between 2008-2013 - GDM was diagnosed according to the diagnosis criteria of the American Diabetes Association - GDM diagnosis is reported as a checkbox item on the birth certificate - maternal exposures to total and speciated PM2.5, nitrogen dioxide (NO2) and ozone (O3) over the 12-week preconception period and trimesters 1 and 2 were estimated based on data from the three closest monitors to each mother's geocoded residential address | - 17,197 women had GDM - An interquartile range (IQR) increase in total PM2.5 exposure was associated with elevated odds for developing GDM over the preconception (adjusted OR = 1.09, 95% CI: 1.06, 1.12), first trimester (OR = 1.13, 95% CI: 1.10, 1.17) and second trimester (OR = 1.13, 95% CI: 1.09, 1.17) periods - Effect estimates increased with adjustment for NO2 and O3 - **We observed modest increases in odds of GDM for IQR increases in first trimester ammonium ion PM2.5 (OR = 1.03, 95% CI: 1.00, 1.05) and sulfate PM2.5 (OR = 1.03, 95% CI: 1.00, 1.05) exposures, as well as preconception Cr PM2 Exposures to PM2.5, before and during pregnancy were associated with elevated odds of GDM.5 exposures (OR = 1.05, 95% CI: 1.02, 1.07)** |
| Robledo et al. 2015 | USA | Human study  Retrospective cohort study | Preconception and early pregnancy **air pollution** exposures and risk of gestational diabetes mellitus | - N= 219,952 pregnant women - Between 2002 – 2008 - obstetric electronic medical records (EMRs) were used - GDM was recorded in the medical record or in discharge records using the International Classification of Diseases | - There were 11,334 cases of GDM (5.2% of the study sample) - **Preconception maternal exposure to NOX (RR=1.09, 95% CI: 1.04, 1.13) and SO2 (RR=1.05, 1.01, 1.09) were associated with increased risk of subsequent GDM** and risk estimates remained elevated for first trimester exposure - Preconception O3 was associated with lower risk of subsequent GDM (RR=0.93, 0.90, 0.96) but risks increased later in pregnancy. - O3 appeared to increase GDM risk in association with mid-pregnancy exposure but not in earlier time windows. |
| Shen et al. 2017 | Taiwan | Human study  Case-control-study | Maternal exposure to **air pollutants** and risk of gestational diabetes mellitus in Taiwan | - N= 6717 GDM cases - N= 6717 controls - Between 2006 – 2013 - GDM was diagnosed according to International Classification of Diseases, Ninth Revision - Maternal exposures to mean daily air pollutant concentration, derived from 76 fixed air quality monitoring stations within the 12-week period prior to pregnancy and during the 1st and 2nd trimesters, were assessed | - exposure of 1 inter-quartile range (IQR) for PM2.5 and SO2 was found to associate with a significantly elevated odds ratio (OR) of GDM at 1.10 (95% confidence interval (CI) 1.03–1.18 and 1.37 (95% CI 1.30–1.45), respectively - Exposures to PM2.5 and SO2 during the 1st and 2nd trimesters were also associated with significantly increased ORs, which were 1.09 (95% CI 1.02–1.17) and 1.07 (95% CI 1.01–1.14) for PM2.5, and 1.37 (95% CI 1.30–1.45) and 1.38 (95% CI 1.31–1.46) for SO2 ) (p<0,05) - **higher pre- and post-pregnancy exposures to PM2.5 and SO2 for mothers were associated with a significantly but modestly elevated risk of GDM** |
| Yao et al. 2020 | China | Human study  Prospective cohort study | Relationship between temporal distribution of **air pollution** exposure and glucose homeostasis during pregnancy | - N= 5427 nondiabetic pregnant women - between 2015 and 2018 - GDM screening was conducted at 24-28 weeks of pregnancy - The data regarding the average exposure to particulate matter (PM), sulfur dioxide (SO2), and ozone (O3) were obtained from the Hefei Environmental Protection Administration | - 1119 (20.6%) women had GDM - prepregnancy exposure to air pollutants was associated with the risk of GDM in the single pollutant model [odds and 95% confidence interval (CI) of GDM for an interquartile range (IQR) increase was 1.24 (1.06–1.45) for PM2.5, 1.42 (1.26–1.59) for PM10, 1.21 (1.10–1.33) for SO2 and1.19 (1.08–1.31) for O3] - The risk of GDM before pregnancy was higher with long-term exposure to high-concentration pollutants compared with the risk in pregnant women who were not exposed to high-concentration pollutants (χ2 = 41.52, p for trend <0.0001); the ORs and 95% CI values for the exposure times of 1, 2, and 3 months were 1.28 (0.96–1.72), 1.52 (1.06–2.19), and 1.69 (1.11–2.57), respectively - **Prepregnancy long-term air pollution exposure was associated with a higher risk of developing GDM by affecting glucose metabolism. The time window of the maximum effect of PM on GDM and glucose metabolism indicators was observed earlier than that of SO2 and O3.** |
| Ye et al. 2020 | China | Human study  Prospective cohort study | The Associations of **Ambient Fine Particulate Matter** Exposure During Pregnancy With Blood Glucose Levels and Gestational Diabetes Mellitus Risk | - N= 967 pregnant women - Between 2013-2016 - According to the GDM criteria of the International Association of Diabetes and Pregnancy Study Group women underwent GDM screening - PM2.5 exposure during pregnancy for each participant was estimated by means of land-use regression models based on each woman’s residential address during pregnancy | - An interquartile-range increase in PM2.5 exposure (33.84 μg/m3 for trimester 1 and 33.23 μg/m3 for trimester 2) was associated with 36% (95% confidence interval (CI): 1.15, 1.61) and 23% (95% CI: 1.01, 1.50) increased odds of GDM during trimester 1 and trimester 2, respectively - An interquartile-range increment of PM2.5 exposure during trimester 1 increased 1-hour and 2-hour blood glucose levels by 1.40% (95% CI: 0.42, 2.37) and 1.82% (95% CI: 0.98, 2.66), respectively - The same increment of PM2.5 exposure during trimester 2 increased fasting glucose level by 0.85% (95% CI: 0.41, 1.29) - **high PM2.5 exposure during pregnancy increases blood glucose levels and GDM risk in Chinese women. (p<0,05)** |
| Yu et al. 2020 | China | Human study  Retrospective cohort study | **Fine particular matter** and its constituents in air pollution and gestational diabetes mellitus | - N= 54,517 pregnant women - Between 2015-2016 - GDM was diagnosed according to the International Association of Diabetes and Pregnancy Study Groups (IADPSG) criteria - Average concentrations of PM2.5 and six constituents (organic matter, black carbon, sulfate, nitrate, ammonium and soil dust) were estimated by using various information from satellite-, simulation- and monitor-based sources | - The incidence of GDM was 10.8% - An interquartile range (IQR) increase in PM2.5 exposure in the 2nd trimester of pregnancy was associated with an increased GDM risk in the single pollutant model, [adjusted odds ratio (aOR) = 1.11 and 95% confidence interval (CI): 1.01–1.22]. - Exposure to organic matter (aOR = 1.14; 95%CI: 1.05–1.23), black carbon (aOR = 1.15; 95%CI: 1.07–1.25) and nitrate (aOR = 1.13; 95%CI: 1.02–1.24) during 2nd trimester were associated with increased risks of GDM - **Exposure to PM2.5 in 2nd trimester of pregnancy was associated with an increased risk of GDM. Organic matter, black carbon and nitrate may be the main culprits for the association**. |
| Zhang et al. 2021 | China | Human study  prospective cohort study | **Ambient air pollution** exposure during pregnancy and gestational diabetes mellitus in Shenyang, China: a prospective cohort study | - N= 2348 pregnant women - GDM was diagnosed according to the 2010 recommendations of the International Association of Diabetes and Pregnancy Study Group - Air pollution exposures of PM10, PM2.5, SO2, NO2, CO, and O3 during the first and second trimesters were estimated from national fixed monitoring stations - Air pollution exposures during the first and second trimesters were estimated by using land-use regression models developed from 31 monitoring stations | - significant associations between prenatal exposure to NOx and SO2 and the development of GDM during the second trimester: the largest effect on GDM was exposure to SO2 (odds ratio (OR): 1.77, 95% confidence interval (CI): 1.23–2.56) in the largest quartile compared with the lowest quartile was found - Significant interactions between age, BMI, parity, sleep quality, and air pollution exposures were observed; stratified analysis showed stronger associations between GDM and high air pollutant exposure in pregnant women with older age, larger BMI, poorer sleep quality, and more parity - **air pollution exposure during the second trimester** **was significantly associated with GDM in a prospective birth cohort study in Northeast China. SO2, oxynitride (NOX, NO2, NO), CO, and O3 all showed a linear trend effect on GDM (p<0,05)** |
| Zhang et al. 2020 | China | Human study  prospective cohort study | **Ambient air pollution** exposure and gestational diabetes mellitus in Guangzhou, China: A prospective cohort study | - N= 5421 pregnant women - Between 2011–2014 - Daily PM2.5, PM10, SO2 and NO2 levels were collected from 10 monitoring stations - GDM was diagnosed According to the criteria for diagnosis of GDM established by the National Health Commission of the People's Republic of China (OGTT) | - The GDM incidence was 11.69% - An interquartile range (IQR) increase in first trimester SO2 was associated with increased GDM risk in the single pollutant model, the adjusted odds ratio (aOR) and 95% confidence interval (CI) was 1.22 (1.02–1.47). - When stratified by maternal folic acid supplementation, first trimester exposure to SO2 was associated with increased GDM risk among women taking folic acid supplements (aOR = 1.25, 95% CI: 1.03–1.53) and P value for interaction was 0.13. - **First trimester exposure to SO2 was associated with increased GDM risk, especially during the 4th to 10th gestational weeks**. |
| Zhang et al. 2020 | China | Human study  Cohort study | Increased risk of gestational diabetes mellitus in women with higher prepregnancy **ambient PM (2.5)** exposure | - N= 11,639 pregnant women - From 2016 – 2017 - A series of validated land-use regression (LUR) models were built to assess individual exposure to PM2.5 in a 1 × 1 km area at both work and home addresses following a time-weighted pattern - GDM was diagnosed by clinicians based on an oral glucose tolerance test (OGTT) | - 2776 (23.85%) women had GDM - Positive associations were observed among the interquartile ranges (IQRs) of exposure to PM2.5 within three months before pregnancy and GDM (OR = 2.61, 95% CI: 1.40–4.93, p < .01) as well as fasting glucose levels (β = 0.57, 95% CI: 0.45–0.68, p < .01). - The diabetogenic effects of PM2.5 gradually increased from the first month before pregnancy, peaked in the second month and then gradually decreased until the third month when the week-specific exposure were analyzed to identify the sensitive time window - **Results confirmed that higher exposure to PM2.5 within three months before pregnancy is significantly associated with increased risk of GDM and elevated fasting glucose levels** |
